# ADAR3 activates NF-κB signaling and promotes glioblastoma cell resistance to temozolomide

**DOI:** 10.1038/s41598-022-17559-4

**Published:** 2022-08-03

**Authors:** Reshma Raghava Kurup, Eimile K. Oakes, Pranathi Vadlamani, Obi Nwosu, Pranav Danthi, Heather A. Hundley

**Affiliations:** 1 Department of Biology, Indiana University, Bloomington, IN 47405 USA; 2 Medical Sciences Program, Indiana University School of Medicine-Bloomington, Bloomington, IN 47405 USA

**Keywords:** Biochemistry, Cancer, Molecular biology

## Abstract

The RNA binding protein ADAR3 is expressed exclusively in the brain and reported to have elevated expression in tumors of patients suffering from glioblastoma compared to adjacent brain tissue. Yet, other studies have indicated that glioblastoma tumors exhibit hemizygous deletions of the genomic region encompassing ADAR3 (10p15.3). As the molecular and cellular consequences of altered ADAR3 expression are largely unknown, here we directly examined the impacts of elevated ADAR3 in a glioblastoma cell line model. Transcriptome-wide sequencing revealed 641 differentially expressed genes between control and ADAR3-expressing U87-MG glioblastoma cells. A vast majority of these genes belong to pathways involved in glioblastoma progression and are regulated by NF-κB signaling. Biochemical and molecular analysis indicated that ADAR3-expressing U87-MG cells exhibit increased NF-κB activation, and treatment with an NF-κB inhibitor abrogated the impacts of ADAR3 on gene expression. Similarly, we found that increased cell survival of ADAR3-expressing cells to temozolomide, the preferred chemotherapeutic for glioblastoma, was due to increased NF-κB activity. Aberrant constitutive NF-κB activation is a common event in glioblastoma and can impact both tumor progression and resistance to treatment. Our results suggest that elevated ADAR3 promotes NF-κB activation and a gene expression program that provides a growth advantage to glioblastoma cells.

## Introduction

Cancer researchers have largely focused on how epigenetic changes and expression of oncogenic transcription factors drive cancer initiation and progression. However, aberrant RNA modification and post-transcriptional gene regulation also contribute to oncogenic gene expression programs^1–3^ and these changes are largely driven by altered expression and/or misregulation of RNA binding proteins^4,5^. In fact, recent consortium efforts to sequence thousands of tumors have identified genes that are recurrently mutated, amplified or deleted in cancer, and specifically for the Catalogue of Somatic Mutations in Cancer, RNA binding proteins make up 5% of these potential oncogenic drivers^6^. Therefore, understanding the role of misregulated RNA binding proteins helps in development of therapeutic strategies.

Adenosine deaminases that act on RNA (ADARs) are a family of RNA binding proteins that can regulate gene expression by binding cellular RNAs and/or catalyzing one of the most abundant RNA modifications in mammals^7,8^. In humans, ADAR1 and ADAR2 are the enzymatically active ADARs that mediate conversion of adenosine (A) to inosine (I). Due to differences in base-pairing, A-to-I RNA editing contributes to gene regulation by influencing processes such as RNA splicing, stability, localization and translation^9,10^. Cancer transcriptomes exhibit altered RNA editing^11,12^, and individual editing events have been shown to both alter tumor suppressor activity^13,14^ and impact oncogene function. Editing by ADAR2 is known to alter genome stability, cell proliferation/migration and other cancer hallmarks, most notably in pediatric astrocytoma^15^ and glioblastoma^16–18^. Glioblastoma (grade IV astrocytoma) is the most aggressive and malignant brain tumor and has a poor patient survival with current treatment strategies including surgery, radiation and chemotherapy^19^. Interestingly, elevated ADAR1 expression was found to correlate with poor survival of glioblastoma patients, and ADAR1 was shown to play a cancer-promoting role in glioblastoma^20^. Importantly, the oncogenic ADAR1 function in glioblastoma is editing-independent, suggesting that ADARs may also play important oncogenic roles as RNA binding proteins.

The third member of the human ADAR family, ADAR3, lacks deaminase activity but has been reported to have both single-stranded and double-stranded RNA binding activity in vitro^21^. Previously, our lab demonstrated that when aberrantly expressed in U87 glioblastoma cells, ADAR3 binds to *GRIA2* pre-mRNA and inhibits ADAR2-mediated editing of one specific adenosine. Editing of that adenosine (referred to as the Q/R site) is essential in mammals^22^ and reduced in several neuropathological diseases, including glioblastoma^15,23^. Consistent with these results, our group^24^ and another independent study^25^ found that ADAR3 protein expression is increased in tumor samples of patients suffering from glioblastoma as compared to tumor-adjacent tissue from the same patient. Earlier studies of approximately 100 different human brain tumors (including both low-grade astrocytomas and glioblastomas) found decreased ADAR3 mRNA expression in brain tumors compared to non-matched normal brain tissue^26^. It is unclear if the differing conclusions on ADAR3 expression in glioblastoma are due to the use of non-matched normal brain tissue or examination of protein versus mRNA levels. In regard to the latter, it was previously observed that the elevated ADAR3 protein expression in glioblastoma tumors did not directly correlate with ADAR3 mRNA expression^25^. In fact, ADAR3 mRNA expression decreased in glioblastoma tumor samples compared to the adjacent, normal tissue^25^, suggesting an inverse relationship between ADAR3 mRNA and protein expression. Decreased ADAR3 mRNA expression compared to normal brain samples was also reported in two recent transcriptome-wide studies of 88–145 glioblastoma tumor RNA-seq datasets obtained from The Cancer Genome Atlas (TCGA) and the Chinese Genome Glioma Atlas studies^27,28^. It is important to note that one of the above studies suggested that chromosomal deletion (hemizygous) of ADAR3 occurs in 85% of glioblastomas^27^, but it is unclear if this deletion is specific to the ADAR3 genic region or a result of the well-established chromosome 10 loss of heterozygosity that is the most frequent genetic abnormality observed in glioblastoma^29–31^.

To directly assess whether ADAR3 impacts the glioblastoma transcriptome to result in oncogenic consequences, herein we performed high-throughput sequencing comparing gene expression of U87-MG (hereafter referred to as U87) glioblastoma cells expressing ADAR3 and control cells. We observed that ADAR3-expressing cells exhibit altered expression of genes regulated by the NF-κB pathway. While NF-κB is most widely known as a master transcription factor^32^ that regulates inflammation and immunity in response to stimuli^33^, aberrant activation of NF-κB has been observed in several types of cancers and can control the expression of genes that promote tumor cell survival and other oncogenic hallmarks^34^.

NF-κB is a family of five transcription factors composed of homodimers and heterodimers of Rel family of proteins—p65 (RelA), p50 (NF-κB1, p105), p52 (NF-κB2 (p52/p100), RelB and c-Rel^32^. NF-κB is held inactive in the cytoplasm^35^, but upon stimulation, NF-κB translocates into the nucleus and regulates expression of target genes^36^. It is important to note that aberrant constitutive NF-κB activation is observed in several cancers and is a common event in glioblastoma^37^. In addition to nuclear localization, phosphorylation of Ser536 in the transactivation domain of the NF-κB p65 subunit plays a significant role in transcriptional activation^38^. Both expression and phosphorylation of p65 at Ser356 are positively correlated with grade of glioma^39^. Thus, NF-κB and its target genes are potential therapeutic targets for glioblastoma^40,41^.

Using subcellular fractionation and analysis of phosphorylated p65, we demonstrate that ADAR3-expressing U87 glioblastoma cells have elevated levels of constitutively activated NF-κB. Upon stimulation, these cells are capable of further gene upregulation, while treatment with an NF-κB inhibitor abrogated the impacts of ADAR3 on gene expression. As increased NF-κB activation mediates radioresistance^41^ and blocks chemotherapy-induced cell death^42^, these phenotypes were examined in ADAR3-expressing glioblastoma cell lines. We found that ADAR3-expressing glioblastoma cells exhibited increased cell survival in response to ionizing radiation and temozolomide, the primary standard of care used to treat glioblastoma patients. Importantly, the increased resistance of ADAR3-expressing glioblastoma cells to temozolomide was not observed when cells were treated with an NF-κB inhibitor. Together, our studies reveal that elevated ADAR3 expression observed in glioblastoma patients impacts oncogenesis by increasing NF-κB driven cellular survival.

## Results

### ADAR3 expression leads to altered expression of NF-κB pathway genes in glioblastoma

**Figure 1:**
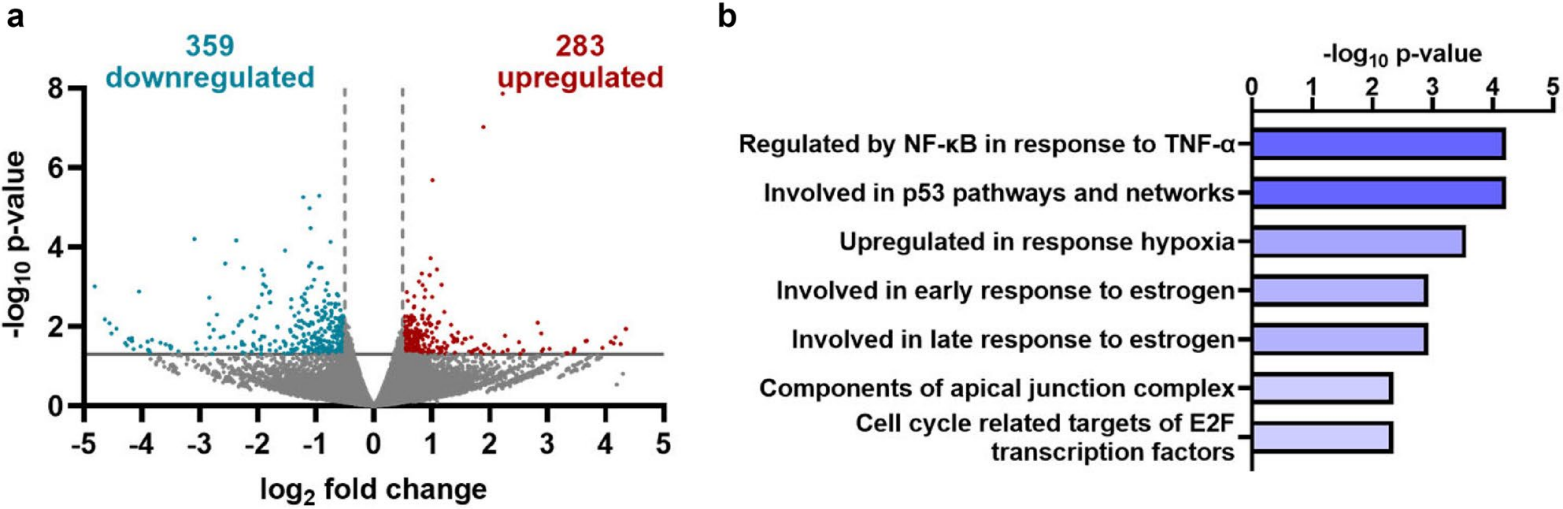
Altered gene expression associated with ADAR3 expression in U87 cells. (**a**) Dots represent individual genes that are downregulated (blue), upregulated (red), or not significantly different (gray) in ADAR3-expressing U87 cells compared to control U87 cells in three biological replicates of RNA-seq data. Negative log_10_ p**-**values of differential expression (y-axis) are plotted against the average log_2_ fold change in expression (x-axis). Genes considered significantly differentially expressed exhibited log_2_ fold change of 0.5 (light gray broken vertical lines) and a p-value of 0.05 [log_10_ p-value of 1.3, light gray horizontal line]. (**b**) Significantly enriched pathways for the 641 differentially expressed genes are listed in descending order of the -log_10_ p-value provided by the Gene Set Enrichment Analysis computational method.

Previous studies from our lab^24^ and others^25^ have indicated that ADAR3 is highly expressed in glioblastoma tumors compared to normal adjacent brain tissue from the same patient. However, whether ADAR3 promotes an oncogenic gene expression program is unknown. As a first step to understanding the impacts of ADAR3 on the glioblastoma transcriptome, we took an unbiased approach of performing RNA-sequencing (RNA-seq). Polyadenylated RNA was isolated from the U87 glioblastoma cell line, which has low ADAR3 expression^24^, and U87 cells transduced with a virus expressing ADAR3 under the control of the CMV promoter, which we have shown previously to provide a high ADAR3 expressing glioblastoma cell line condition^24^. Differential expression analysis identified 641 significantly differentially expressed genes (excluding ADAR3) from three biological replicates of RNA-seq data (p < 0.05, log_2_fold change >|0.5|) (Fig. 1, Supplementary Table 1). The differentially expressed genes are nearly equally split between up- (44%) and downregulated (56%) genes (Fig. 1a). To validate the RNA-seq findings, five genes identified as either up- or downregulated were randomly chosen and analyzed by quantitative real-time PCR (qRT-PCR) in three independent biological replicates of RNA isolated from control and ADAR3-expressing U87 cells. Consistent with the RNA-seq data, all five genes (*TCFL5*, *BLM*, *CD22*, *INKA2* and *PFKFB4*) followed the predicted direction of up- or downregulation between ADAR3-expressing and control cells; however, it should be noted that only four of the five genes were found to be significantly differentially expressed by qRT-PCR (Supplementary Fig. S1a).

Molecular signatures of the ADAR3-mediated differentially expressed genes were investigated using Gene Set Enrichment Analysis (GSEA) (MSigDB database version 7.1)^43^. Using the hallmark gene sets for GSEA^44^, the 641 genes differentially expressed between control and ADAR3-expressing cells were found to have significant enrichment with gene sets known to be regulated by NF-κB signaling and hypoxia as well as those involved in the p53 pathway, and several other gene sets (Fig. 1b). Similar GSEA of 10 random groups of 641 genes expressed in the ADAR3-expressing U87 cells did not reveal significant enrichment for any of these gene sets. When GSEA was performed on the up- and downregulated genes separately, the genes regulated by NF-κB signaling and hypoxia as well as those involved in the p53 pathway were found to be significantly enriched specifically in the upregulated genes (Supplementary Fig. S1b). In addition, as seen with the overall dataset, genes defining both the early and late response to estrogen as well as genes encoding cell cycle related targets of E2F transcription factors were significantly enriched in the downregulated genes (Supplementary Fig. S1c).

Together, these data indicate that many genes differentially expressed upon ADAR3 expression belong to pathways involved in glioblastoma progression. For example, p53 signaling pathways facilitate increased glioma survival by activation of DNA repair genes and improved drug resistance^45,46^, and upregulation of the hypoxic response contributes to tumor progression by enhancing glioma stem cell maintenance^47^, radioresistance^48^, angiogenesis^49^ and metabolic programming^50^. Furthermore, the categories of up- and downregulated genes suggest that ADAR3 expression promotes oncogenic gene expression programs and reduces expression of tumor suppressive pathways. For example, the estrogen response gene set was enriched amongst the downregulated genes (Supplementary Fig. S1c), and both epidemiological^51^ and recent experimental evidence indicate that estrogens and transcription factors that respond to these ligands function as tumor suppressors in glioblastoma^52,53^. For oncogenic gene expression programs potentially driven by ADAR3, it is important to note that amongst all the differentially regulated genes, the most enriched gene set was the TNF-α induced NF-κB pathway (Fig. 1b), which is known to regulate several other enriched pathways identified in our analysis, particularly the upregulated genes^54^. For example, NF-κB activation leads to MDM2 upregulation and thus p53 destabilization, which in turn blocks chemotherapy-induced cell death^42^. Based on these data, ADAR3-expressing cells exhibit several key alterations to the glioblastoma transcriptome that may provide cellular growth advantages.

### ADAR3 expression results in increased phosphorylation and nuclear localization of NF-κB

**Figure 2:**
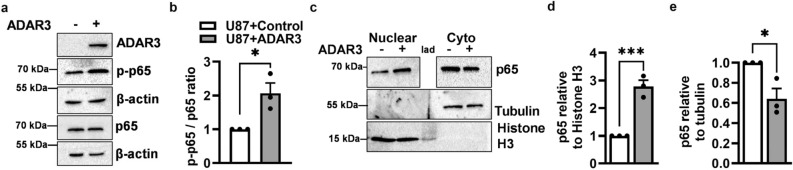
Increased phosphorylation and nuclear localization of the NF-κB p65 subunit upon ADAR3 expression. (**a**) Control (ADAR3−) and ADAR3-expressing (ADAR3+) U87 cells were lysed and subjected to immunoblotting with antibodies against ADAR3, p65, phosphorylated S356 p65 (p-p65), and β-actin. As phosphorylated and unphosphorylated p65 migrate at the same position on SDS-PAGE, expression of p-p65 and p65 was examined on independent immunoblots, with control detection of β-actin performed for each immunoblot. Blot is a representative image (replicate 2) of three biological replicates of the p65 and p-p65 immunoblots and one replicate of the ADAR3 immunoblot. Uncropped blot images are included in the supplementary information file. (**b**) Quantification of phosphorylated p65 protein (p-p65) to total p65 protein levels was determined relative to β-actin controls for each immunoblot. The relative p-p65/p65 ratios were normalized to the control cell line. Error bars represent the standard error of the mean (SEM) for three biological replicates. Statistical significance was determined using a two-tailed unpaired t-test. *p ≤ 0.05 (**c**) Cell equivalent amounts of nuclear and cytoplasmic fractions of control and ADAR3-expressing U87 cells were subjected to immunoblotting using antibodies against p65, tubulin and Histone H3. To determine the accuracy of the subcellular fractionation, the entire immunoblot was probed with Tubulin and Histone H3 antibodies. As cytoplasmic levels of p65 are significantly higher than nuclear p65 levels, to allow for exposure in the dynamic range and proper quantification, the entire p65 blot was incubated with primary antibody and exposed to capture the cytoplasmic p65 levels. Subsequently, the nuclear p65 immunoblot was cropped away from the cytoplasmic p65 samples and re-subjected to enhanced chemiluminescence detection for detection within the dynamic range. Blot is a representative image (replicate 1) of three biological replicates and uncropped blot images are included in the supplementary information file. (**d**,**e**) Quantification of the p65 level relative to Histone H3 in nuclear (**d**) and tubulin in cytoplasmic (**e**) fractions were normalized to the control cell line for three biological replicates. Error bars represent SEM. Statistical significance was determined by a two-tailed unpaired t-test. *p ≤ 0.05, ***p ≤ 0.0005.

As NF-κB is a master regulator of many tumor-promoting pathways and a potential therapeutic target in glioblastoma^40,55^, the ADAR3-NF-κB axis was further explored. To test whether ADAR3 expression results in increased NF-κB activation, the expression and post-translational processing of p65 and p52/p100, key components of the canonical and non-canonical NF-κB pathways, respectively^56^, were examined. Specifically, the expression and phosphorylation at Serine 536 (S536) in the p65 subunit, a marker for activation of NF-κB, was measured in protein lysates from ADAR3-expressing cells and compared to control U87 cells. Immunoblotting analysis did not detect any significant change in the expression of the NF-κB p65 subunit (Fig. 2a), which is consistent with the observation that p65 (*RELA*) was not misregulated at the mRNA level in the transcriptome-wide differential gene expression analysis (Supplementary Table 1). However, an increase in the phosphorylation of the p65 subunit upon ADAR3 expression was observed (Fig. 2a). It should be noted that the control U87 cells express some phosphorylated p65 (Fig. 2a), which is consistent with previous studies indicating U87 cells exhibit constitutive NF-κB activity^57^. To determine whether the phosphorylated p65 increase in the ADAR3-expressing U87 cells was significant, quantitative immunoblotting was performed. This analysis revealed an approximate twofold increase in S356 p65 phosphorylation in ADAR3-expressing cells compared to control U87 cells (Fig. 2b). These data suggest that ADAR3-expressing glioblastoma cells have a significant increase in canonical NF-κB activation. To more directly test whether ADAR3-expressing cells exhibited activation of the non-canonical NF-κB pathway, immunoblot analysis was performed to examine the expression and processing of p100 precursor protein to form the active p52 subunit of the non-canonical NF-κB pathway (Supplementary Fig. S2a). In both control and ADAR3-expressing U87 cells, p52 expression was barely detectable and the expression of p100 remained unaltered between control and ADAR3-expressing U87 cells (Supplementary Fig. S2a), suggesting ADAR3 expression did not lead to activation of the non-canonical NF-κB pathway.

To delve further into the mechanism of how ADAR3 expression resulted in increased NF-κB activation, nuclear translocation of the NF-κB p65 subunit was monitored by performing subcellular fractionation followed by quantitative immunoblotting. Accuracy of the fractionation was determined by performing immunoblots on the nuclear and cytoplasmic fractions and probing for Tubulin (cytoplasmic marker) and Histone H3 (nuclear marker) (Fig. 2c). Due to the high levels of cytoplasmic p65 in U87 cells, changes in p65 subcellular localization were quantified independently. Using the nuclear fraction of control and ADAR3-expressing U87 cells, the amount of the p65 subunit of NF-κB was quantified relative to a nuclear protein, Histone H3. A significant (approximately threefold) increase in nuclear localization of the NF-κB p65 subunit was observed in ADAR3-expressing U87 cells compared to control cells (Fig. 2c,d). Similarly, p65 levels in the cytoplasmic fraction were significantly reduced in ADAR3-expressing U87 cells (~ 50%) compared to control cells (Fig. 2c,e). Together these data suggest that ADAR3-expressing cells have increased p65 nuclear localization, which is indicative of activation of the canonical NF-κB pathway. Canonical NF-κB activation is regulated by IκB-α levels, which in turn are regulated by IκB kinase (IKK)-mediated phosphorylation of IκB-α at Ser32 and Ser36 which results in IκB-α proteasomal degradation and entry of phosphorylated p65 into the nucleus^58^. To further test whether ADAR3 expression results in activation of the canonical pathway, expression and Ser32/Ser36 phosphorylation of IκB-α was examined. As these phosphorylation events result in IκB-α degradation, the control and ADAR3-expressing cells were pretreated with the proteasomal inhibitor MG132 for 4 h before lysate preparation. Immunoblot analysis revealed an increase in the ratio of phosphorylated IκB-α to total IκB-α in ADAR3-expressing U87 cells compared to control cells (Supplementary Fig. S2b). Together, these data indicate that ADAR3-expressing cells have elevated canonical NF-κB signaling leading to increased NF-κB p65 phosphorylation and p65 nuclear localization.

### NF-κB activation is required for the effects of ADAR3 on the glioblastoma transcriptome

**Figure 3:**
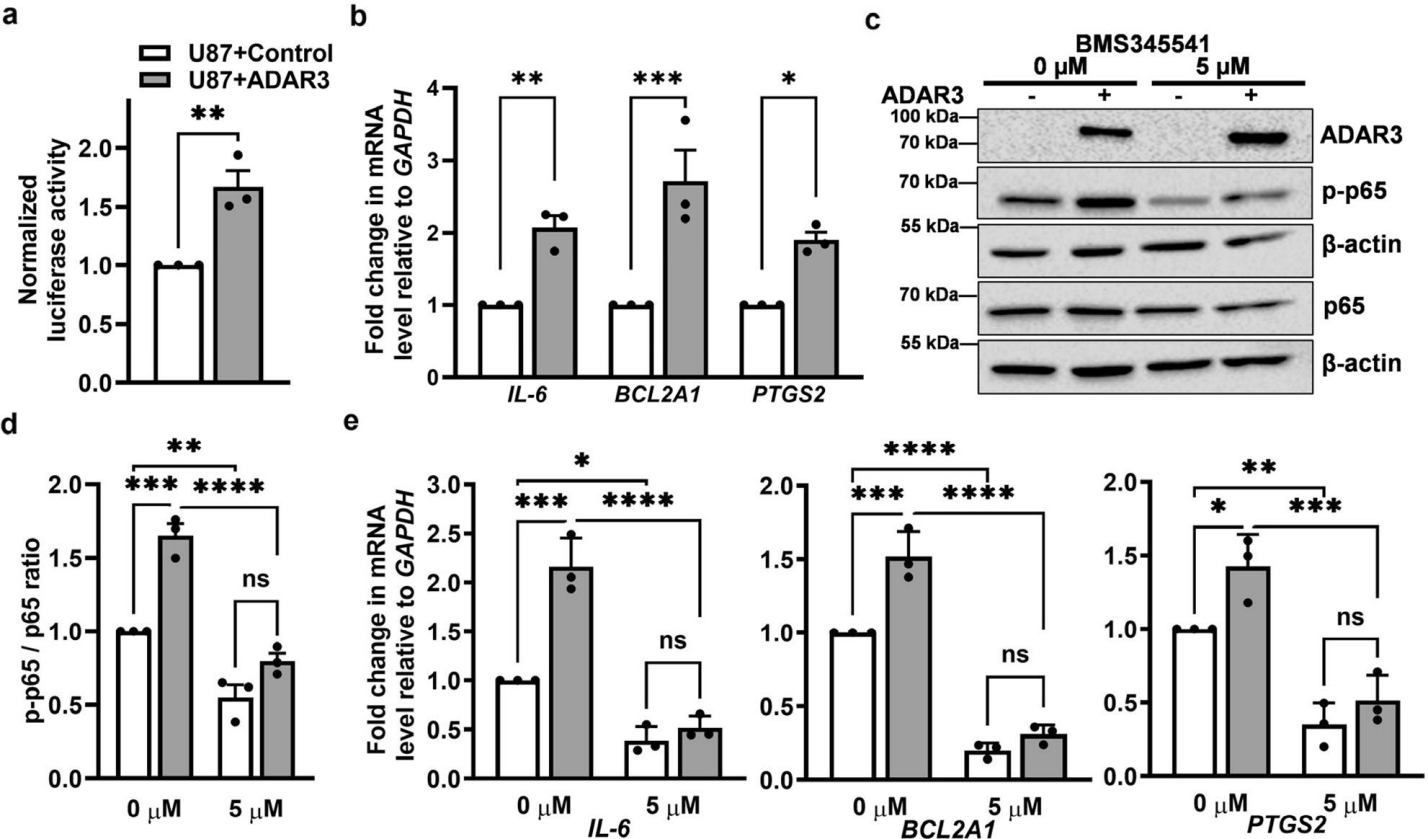
NF-κB-dependent differential gene expression in ADAR3-expressing U87 cells. (**a**) Control and ADAR3-expressing U87 cell lines were co-transfected with pNF-κB-Luc and pRenilla-Luc. Firefly and *Renilla* luciferase activity was determined after 24 h (h). The ratio of firefly to *Renilla* luciferase was calculated. The luciferase activity ratio in ADAR3-expressing cells was normalized to that of control cells for three biological replicates. Error bars represent SEM. Statistical significance was determined using a two-tailed unpaired t-test. **p ≤ 0.005 (**b**) qRT-PCR quantification of the level of the indicated NF-κB target genes relative to *GAPDH* and normalized to the control U87 cell line. The mean of three biological replicates is plotted with SEM. Statistical significance was determined using a two-way ANOVA Sidak's multiple comparisons test. *p ≤ 0.05, **p ≤ 0.005 ***p ≤ 0.0005 (**c**) Control and ADAR3-expressing U87 cells were treated with 0 (DMSO only) or 5 μM of BMS345541 for 24 h. An equivalent amount of cell lysates was subjected to quantitative immunoblotting with antibodies against ADAR3, p65, phosphorylated S356 p65 (p-p65), and β-actin. Blot is a representative image (replicate 1) of three biological replicates and uncropped images are included in the supplementary information file. (**d**) The ratio of p-p65 to total p65 relative to β-actin was quantified and normalized to U87 control cells with 0 μM BMS345541 treatment. The mean of three biological replicates is plotted with error bars representing SEM. Statistical significance was determined using two-way ANOVA Tukey’s multiple comparisons test. **p ≤ 0.005 ***p ≤ 0.0005, ****p < 0.0001, ns indicates no significant difference. (**e**) qRT-PCR quantification of the level of the indicated NF-κB target genes relative to *GAPDH* in the indicated cells after treatment with 0 or 5 μM BMS345541 for 24 h. The relative values were normalized to the control cell line with 0 μM BMS345541 treatment. The mean of three biological replicates is plotted with SEM. Statistical significance was determined by two-way ANOVA Tukey's multiple comparisons test. *p ≤ 0.05, **p ≤ 0.005 ***p ≤ 0.0005, ****p < 0.0001, ns indicates no significant difference.

To directly test whether the increased phosphorylation and nuclear localization of p65 in ADAR3-expressing U87 cells alters downstream NF-κB-dependent gene regulation, activation of a reporter controlled by the NF-κB promoter was analyzed. In this assay, a plasmid with firefly luciferase transcription under the control of the NF-κB consensus promoter sequence was co-transfected with a plasmid where *Renilla* luciferase was expressed via a constitutively active promoter. Luciferase activity was measured in lysates of ADAR3-expressing and control U87 cells that were transiently co-transfected with both plasmids. Consistent with elevated NF-κB activation, ADAR3-expressing U87 cells exhibited significantly increased firefly luciferase activity compared to control cells (Fig. 3a).

To further determine if the increased NF-κB activity observed in ADAR3-expressing U87 cells leads to downstream gene expression changes, we sought to monitor endogenous expression of NF-κB target genes after treatment with a known inhibitor of NF-κB signaling. As a first step, qRT-PCR was used to determine endogenous expression of three NF-κB target genes, *IL-6*, *BCL2A1*, *PTGS2*, identified from the GSEA analysis (Fig. 1b). In basal conditions, all three NF-κB target genes exhibited significantly increased expression in ADAR3-expressing U87 cells compared to control cells (Fig. 3b). To determine whether the downstream gene expression changes observed in ADAR3-expressing cells were due to NF-κB activation, cells were treated with BMS345541, a highly selective and irreversible inhibitor of IκB kinase (IKK)^59^. IκB proteins bind to NF-κB in the cytoplasm; however, upon phosphorylation by IKK, IκB proteins are degraded and NF-κB is able to translocate to the nucleus to activate transcription^32^. To attempt to inhibit NF-κB activation, control and ADAR3-expressing U87 cells were treated with 5 µM BMS345541 dissolved in dimethyl sulfoxide (DMSO) or DMSO alone for 24 h. Phosphorylation of NF-κB p65 was examined to confirm the inhibition of NF-κB (Fig. 3c). Three independent biological replicates were performed and a significant decrease in the ratio of phosphorylated p65 to total p65 was observed for both control and ADAR3-expressing cells upon BMS345541 treatment (Fig. 3c,d). Importantly, the decreased ratio of phosphorylated p65 to total p65 observed upon BMS345541 treatment was not due to a decrease in ADAR3 expression upon drug treatment (Fig. 3c). Furthermore, consistent with our initial experiments, prior to treatment, ADAR3-expressing cells had significantly higher levels of phosphorylated p65 compared to control cells (Fig. 3c,d). The ability of ADAR3-expressing glioblastoma cells to have elevated NF-κB activation was examined in a second glioblastoma cell line, U118. Similar to the approach described for the U87 cell line, U118 cells were transduced with a retrovirus where ADAR3 expression was driven by the CMV promoter and stable ADAR3-expressing U118 cells were selected using a neomycin resistance gene present in the retrovirus. A control U118 cell line expressing the same resistance gene and CMV promoter but lacking a downstream gene was also obtained. Consistent with the observations in U87 cells, ADAR3-expressing U118 cells exhibit significantly increased phosphorylated p65 expression compared to control cells (Supplementary Fig. S3a,b). Furthermore, inhibition of IKK by 5 µM BMS345541 treatment led to a reduction in the ratio of phosphorylated p65 to total p65 in control and ADAR-expressing U118 cells (Supplementary Fig. S3a,b), without a reduction in ADAR3 expression (Supplementary Fig. S3a). Together, these data indicate that ADAR3-expressing glioblastoma cells have increased activation of the NF-κB pathway and suggest that ADAR3 acts upstream of IKK.

Upon BMS345541 treatment, we observed a significant reduction in the expression of NF-κB target genes (*IL-6*, *BCL2A1*, and *PTGS2*) for both control and ADAR3-expressing cells (Fig. 3e). Consistent with our initial analysis, all three NF-κB target genes were upregulated in ADAR3-expressing cells compared to control cells in the 0 µM BMS345541 (Fig. 3e). However, upon treatment with 5 µM BMS345541, there was no longer a significant difference in gene expression between control and ADAR3-expressing cells (Fig. 3e). Together, these data indicate that ADAR3-expressing cells have elevated NF-κB activity, which in turn results in altered downstream gene expression.

### ADAR3-mediated activation of NF-κB results in altered response to stimuli

**Figure 4:**
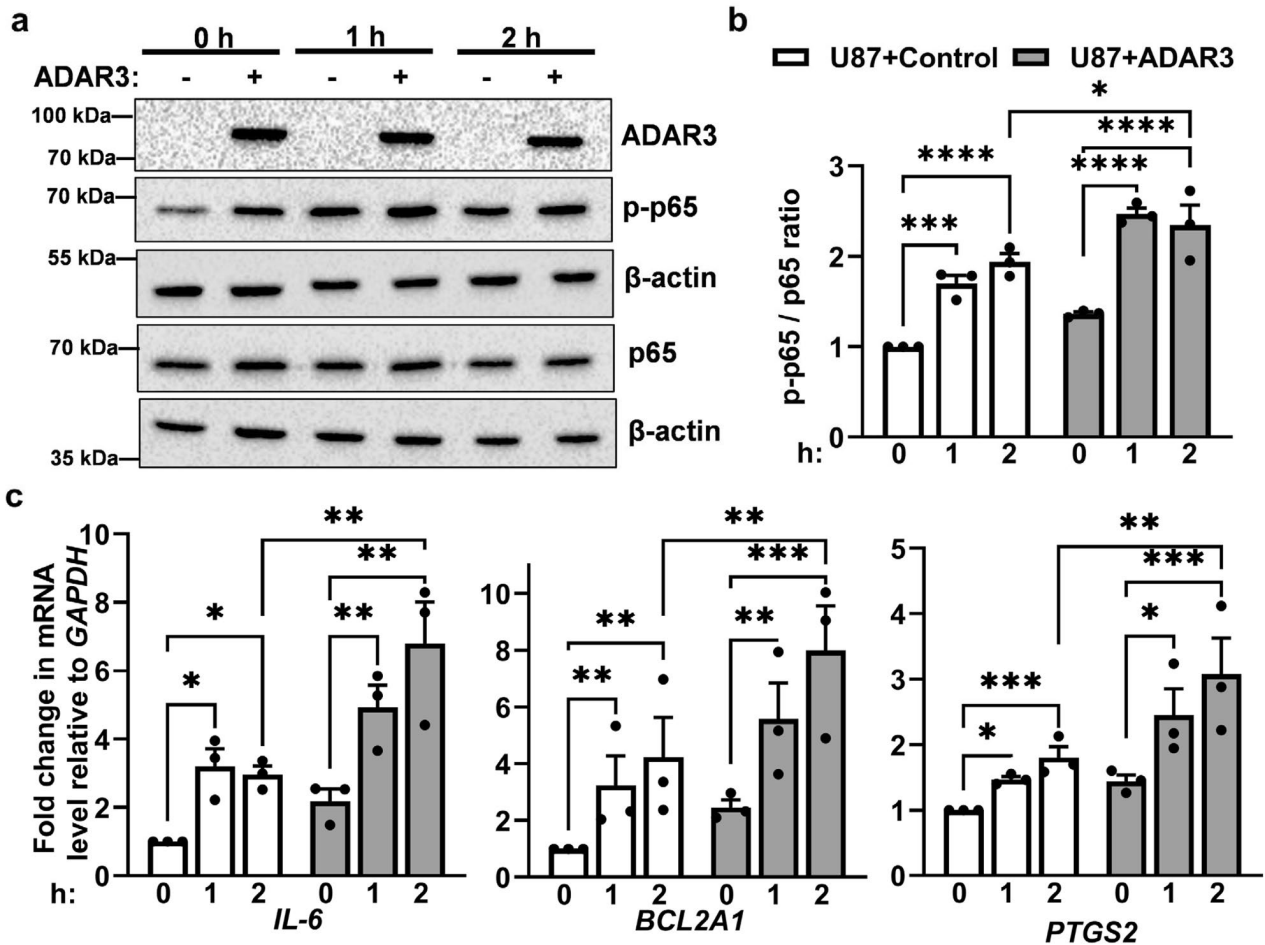
Increased TNF-α induced NF-κB activation in ADAR3-expressing cells. (**a**) Control and ADAR3-expressing U87 cells were treated with 10 ng/mL TNF-α for 0, 1, and 2 h. Equivalent amounts of cell lysate were subjected to immunoblotting with the indicated antibodies. Blot is a representative image (replicate 1) of three biological replicates and uncropped images are included in the supplementary information file (**b**) Quantification of phosphorylated p65 protein (p-p65) to total p65 protein levels was determined relative to β-actin controls for each immunoblot. The relative p-p65/p65 ratios were normalized to the U87 control cells at 0 h. Error bars represent SEM. Statistical significance was determined by two-way ANOVA Fisher’s least significance difference (LSD) test. *p ≤ 0.05, **p ≤ 0.005 ***p ≤ 0.0005, ****p < 0.0001 (**c**) qRT-PCR was performed to quantify the expression of the indicated genes after 0, 1 and 2 h TNF-α treatment. The expression of each gene relative to *GAPDH* in all conditions was normalized to the expression of same gene relative to *GAPDH* in control cells at 0 h. The mean of three biological replicates is plotted with error bars representing SEM. Statistical significance was determined using ordinary two-way ANOVA Fisher’s LSD test. *p ≤ 0.05, **p ≤ 0.005 ***p ≤ 0.0005, ****p < 0.0001, ns indicates no significant difference.

Our data indicate that ADAR3-expressing U87 cells exhibit constitutively elevated NF-κB activation, which is a common event in glioblastoma. As activation of NF-κB signaling occurs in response to a wide-range of stimuli, aberrant NF-κB activation can result from numerous genetic and cellular events. To test whether ADAR3-expressing cells could exhibit further NF-κB activation in response to stimuli, phosphorylation of the p65 subunit of NF-κB as well as downstream gene expression changes were examined after treatment with TNF-α, a proinflammatory cytokine and potent activator of NF-κB signaling^60^. Specifically, ADAR3-expressing and control U87 cells were treated with 10 ng/mL of TNF-α for 2 h. Using quantitative immunoblotting, the ratio of phosphorylated p65 to total p65 expressed was assessed at 0, 1 and 2 h after TNF-α treatment. A significant increase in the p-p65/p65 ratio was observed in U87 control cells one hour after TNF-α treatment and this upregulation remained constant 2 h after treatment (Fig. 4a,b). ADAR3-expressing U87 cells showed a similar trend, with a significant increase in the p-p65/p65 ratio one hour after TNF-α treatment and constant upregulation 2 h after treatment (Fig. 4a,b). Additionally, the levels of phosphorylated p65 after TNF-α treatment were significantly higher in ADAR3-expressing cells compared to control U87 cells (Fig. 4b).

To determine whether the elevated p65 phosphorylation in ADAR3-expressing U87 cells also reflected increased expression of NF-κB target genes, expression of three of these target genes (*IL-6, BCL2A1, PTGS2*) was assessed by qRT-PCR using the same TNF-α treatment regimen described above. For the control U87 cells, a significant increase in expression of all three genes was observed one hour after TNF-α treatment and this upregulation remained constant two hours after treatment (Fig. 4c). For ADAR3-expressing cells, a significant increase in gene expression was also observed one hour after TNF-α treatment (Fig. 4c). Additionally, for the ADAR3-expressing cells, expression of all three genes 2 h after TNF-α treatment was higher than one hour after treatment but this was not considered a statistically significant increase across the three biological replicates. However, it is important to note that at 2 h post TNF-α treatment, the ADAR3-expressing cells exhibit significantly higher expression of all three NF-κB target genes compared to control U87 cells (Fig. 4c). Together, these data indicate that in addition to constitutively elevated NF-κB activation, ADAR3-expressing U87 cells have enhanced NF-κB activation and downstream gene expression in response to TNF-α treatment.

To determine if the ability of ADAR3-expressing U87 cells to respond to NF-κB activating stimuli was limited to TNF-α, similar experiments were performed with the dsRNA mimic polyinosinic-polycytidylic acid (poly I:C), which is used to boost immune activation and immune cell infiltration to improve the efficiency of immunotherapies for solid tumors^61^. Using qRT-PCR, expression of four genes (*IL-6*, *CXCL10*, *TNF-α* and *OAS1*) known to be induced by NF-κB activation after poly I:C treatment^62^ were examined before and after transfection. Consistent with the increased NF-κB activation observed in response to TNF-α treatment, both control and ADAR3-expressing U87 cells exhibited a significant increase in expression of all four NF-κB target genes after polyI:C treatment (Supplementary Fig. S4). Furthermore, the ADAR3-expressing cells exhibited higher overall levels of gene expression than the control cells (Supplementary Fig. S4). However, the exact time after treatment for the elevated response of ADAR3-expressing cells compared to control cells differed. In addition, statistically significant differences were limited to *IL-6* expression at both 16 and 24 h and *TNF-α* expression only at 16 h after poly I:C addition (Supplementary Fig. S4). Together, these data suggest that in addition to constitutive NF-κB activation, ADAR3-expressing U87 cells also exhibit an elevated response to NF-κB activating stimuli.

### ADAR3 expression increases temozolomide resistance of glioblastoma cells

**Figure 5:**
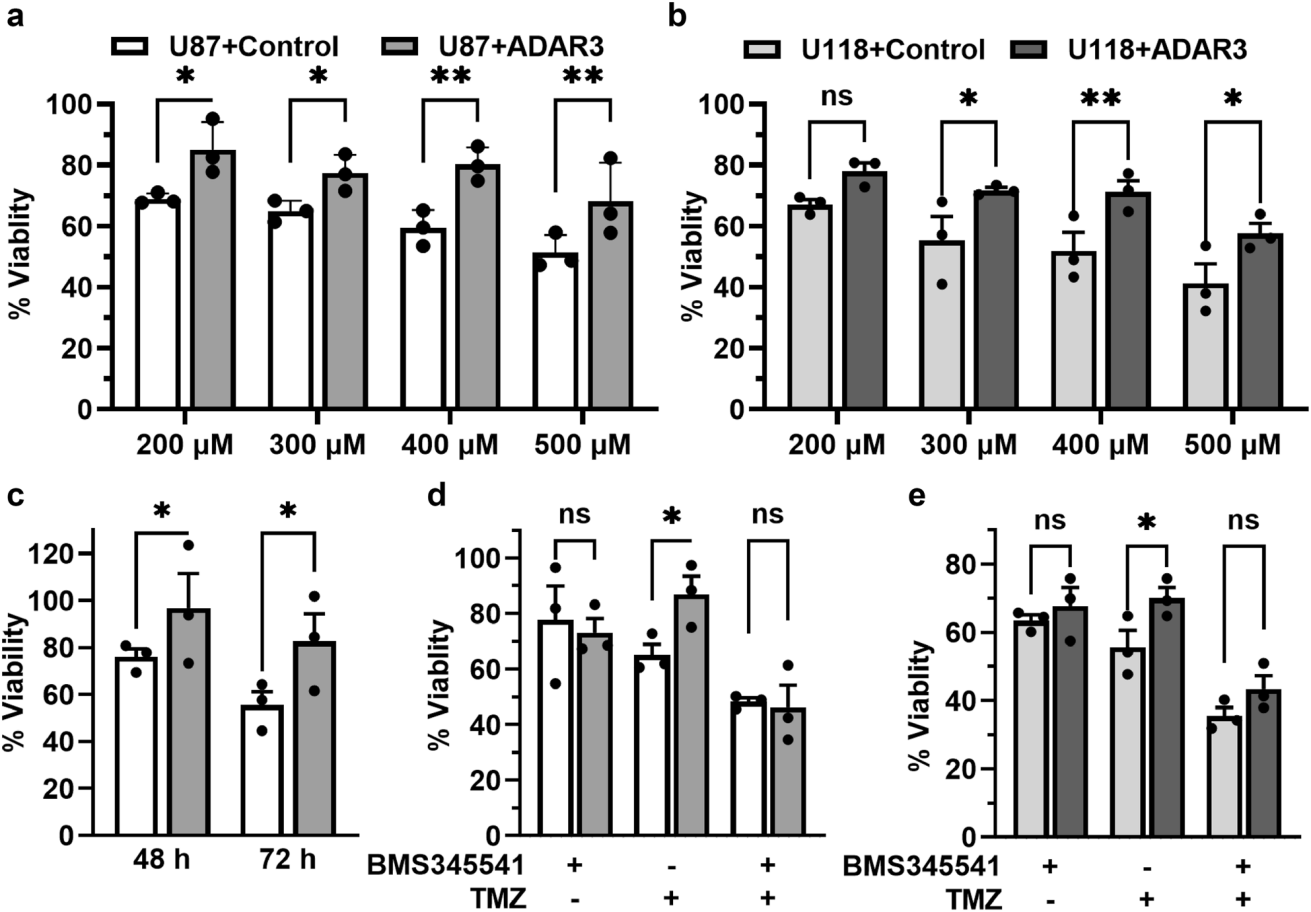
ADAR3 expression increases glioblastoma cell survival to temozolomide and irradiation. (**a**,**b**) Cell viability of ADAR3-expressing U87 (**a**) and U118 (**b**) cells compared to the respective control cells was determined using an MTT assay. Cells were treated with DMSO or the indicated concentration of temozolomide (dissolved in DMSO) for 48 h. Absorbance was measured at 600 nm after incubation with MTT reagent. The absorbance obtained at 0 h for each sample was subtracted from the 48 h readings and live cells in the TMZ treated cells are represented as the percentage of corresponding DMSO treated cells at each time point. (% Cell viability = [A_600_ (t) − A_600_ (0)]_treatment_/[A_600_ (t) − A_600_ (0)]_control_). The mean of three biological replicates is plotted with error bars representing SEM. Statistical significance was determined using two-way ANOVA Fisher’s LSD test. *p ≤ 0.05, **p ≤ 0.005. (**c**) Cell viability of ADAR3-expressing U87 and control U87 cells was determined 48 and 72 h after 5 Grays of gamma irradiation using the MTT assay and formula described above. Statistical significance was determined using two-way ANOVA Fisher’s LSD test. *p ≤ 0.05 (**d**,**e**) Cell viability of control and ADAR-expressing U87 (**d**) and U118 (**e**) cells at each treatment condition was determined using MTT assay as mentioned above. Cells were incubated with DMSO, 400 μM of temozolomide, 5 µM BMS345541 or both temozolomide and BMS345541 for 24 h. The mean of three biological replicates is plotted with error bars representing SEM. Statistical significance was determined using two-way ANOVA Fisher’s LSD test. *p ≤ 0.05.

Glioblastoma is the most aggressive form of brain tumor and responds poorly to the standard-of-care therapies of surgery, irradiation and temozolomide (TMZ)^63^. Increased NF-κB activity is associated with TMZ-resistance in glioma^64^, radioresistance of glioblastoma stem cells^41^, and inhibition of NF-κB activity in combination with temozolomide significantly improved glioma treatment outcome^65^. As our results indicate that ADAR3 expression increases NF-κB activity, we sought to determine whether ADAR3 expression provides glioblastoma cells protection against chemotherapy and/or irradiation. First, ADAR3-expressing and control U87 cells were treated with a concentration gradient (200–500 μM) of temozolomide (dissolved in DMSO) or DMSO as a control. Using an MTT assay, cell viability was determined prior to treatment (0 h) and both 24 and 48 h after addition of TMZ. ADAR3-expressing cells showed increased survival across all TMZ concentrations compared to control cells at both 24 (Supplementary Fig. S5a) and 48 h (Fig. 5a). To determine whether chemoresistance is a common cellular consequence of ADAR3 overexpression, control and ADAR-expressing U118 cells were also tested for survival in response to TMZ treatment. Consistent with our observations from U87 glioblastoma cells, the control U118 cells showed more sensitivity to TMZ treatment compared the ADAR3-expressing U118 cells across the entire concentration range (200–500 μM) with the most significant growth differences occurring at 400 and 500 μM treatment for 24 h (Supplementary Fig. S5b) and 300–500 μM treatment for 48 h (Fig. 5b). In addition to TMZ treatment, cell viability of ADAR3-expressing U87 cells and control U87 cells were examined after treatment with 5 Grays of gamma irradiation. Similar to the chemoresistance phenotype, the ADAR3-expressing U87 cells had increased viability to irradiation compared to the control cells (Fig. 5c). These data suggest that elevated ADAR3 expression in glioblastoma contributes to temozolomide and irradiation resistance.

To further understand the contribution of ADAR3-mediated NF-κB activation in temozolomide resistance, the impact of NF-κB inhibition of TMZ sensitivity of ADAR3-expressing cells was examined. First, the effect of the 24 and 48 h treatment of NF-κB inhibitor BMS345541 on cell viability of control and ADAR3-expressing U87 cells was determined using an MTT assay. The inhibition of NF-κB activity resulted in a slight reduction in survival of both control and ADAR3-expressing cells at 24 h with 60–80% viability (Supplementary Fig. S5c). However, BMS345541 treatment for 48 h was extremely cytotoxic with only 10–30% of cells surviving. For further investigation of the role of NF-κB activity in TMZ resistance of ADAR3-expressing cells, the 400 µM TMZ which showed significant difference in cell viability at 24 h for both U87 and U118 cells was used (Supplementary Fig. S5a,b). While nearly 30–40% of the control cells died between 24 and 48 h hours after 400 µM TMZ addition (Fig. 5a, Supplementary Fig. S5a), the ADAR3-expressing U87 cells were significantly more viable, with nearly 100% of cells surviving 24 h after treatment (Supplementary Fig. S5a) and 80% viability at 48 h after treatment (Fig. 5a). Similarly, the ADAR3-expressing U118 cells had significantly higher cell viability (70–80%) at both 24 and 48 h post TMZ treatment compared to the control cells (approximately 40–60% survival) (Fig. 5b, Supplementary Fig. S5b). U87 and U118 control and ADAR3-expressing cells were treated with NF-κB inhibitor BMS345541 alone and in combination with 400 µM TMZ for 24 h. The inhibition of NF-κB resulted in no significant changes in viability of ADAR3-expressing cells compared to control cells (Fig. 5d,e). Consistent with the previous results (Supplementary Fig. S5a,b), ADAR3-expressing U87 and U118 cells were significantly more resistant to TMZ treatment compared to control cells (Fig. 5d,e). However, treatment of the cells with a combination of BMS345541 and TMZ lead to a decrease in viability of ADAR3-expressing cells compared to the TMZ alone treatment. Moreover, survival of the ADAR3-expressing cells was not significantly different than control cells upon co-treatment of BMS345541 and TMZ (Fig. 5d,e). Together, these data indicate that ADAR3-expressing glioblastoma cells have elevated NF-κB activation that promotes cell survival to the standard of care chemotherapy, temozolomide.

## Discussion

In this study, we have provided the first molecular interrogation of how elevated ADAR3 expression impacts the glioblastoma transcriptome and cell survival. Consistent with the elevated expression observed in glioblastoma tumors and an oncogenic function, our studies revealed that ADAR3 expression in glioblastoma cells leads to elevated NF-κB activation which can confer both downstream gene expression changes and cellular consequences, including increased resistance to temozolomide. Consistent with previous results, the U87 glioblastoma cell line exhibited constitutive NF-κB activation^57^ and the presence of ADAR3 led to a significant increase in the phosphorylation and nuclear localization of the p65 subunit of NF-κB compared to control U87 cells. However, there was no significant change in p65 expression at the mRNA or protein level, suggesting that ADAR3 could indirectly activate NF-κB activity by altering the expression or activity of one of the many upstream factors that regulate NF-κB activity. As inhibition of IKK with BMS345541 attenuated the increased phosphorylation of p65 in both ADAR3-expressing U87 and U118 cells as well as abrogated the enhanced survival of these cells to temozolomide, our data suggest that ADAR3 is likely acting upstream of IKK. However, the exact molecular target of ADAR3 remains to be determined. Interestingly, in our studies, we noticed that treatment of cells with BMS345541 resulted in elevated ADAR3 protein expression, suggesting the presence of a negative feedback loop for ADAR3 expression and NF-κB activation. However, as ADAR3 transcription is controlled by a heterologous promoter in these cell lines, future work should examine this effect in primary glioblastoma cell lines with elevated ADAR3 expression.

It is well established that constitutive NF-κB activation occurs in malignant cells and the tumor microenvironment of most cancers is only rarely due to NF-κB genetic alterations^66^. In glioblastoma, loss of several factors, including PTEN, NF1 and KLF6, as well as increased activity/expression of others, including PIN1, MLK4 and microRNA-30e, have all been shown to contribute to NF-κB activation^54^. For the protein-coding genes listed here, the ADAR3-expressing cells do not show altered mRNA expression in our transcriptome-wide RNA-seq dataset. However, it is possible that ADAR3 binds these mRNAs and regulates protein expression. Similarly, as ADARs in other systems are known to alter small RNA processing pathways through dsRNA binding^9^, it is possible that RNA binding by ADAR3 could influence miRNA levels in glioblastoma. Future studies should focus both on identifying ADAR3-bound target RNAs in glioblastoma cells as well as alterations in small RNA levels that occur upon ADAR3 expression.

An alternative possibility is that ADAR3 inhibits RNA editing, which in turn allows cellular RNAs to engage dsRNA sensors and aberrantly activate the immune response and NF-κB activation. Previous studies from our lab have indicated that ADAR3 binds to dsRNA and inhibits editing of a specific adenosine in *GRIA2* mRNA^24^. However, editing of *GRIA2* occurs on the pre-mRNA; thus, the mature transcript lacks the duplex structure, and the unedited transcript is unlikely to engage dsRNA sensors. To date, a global approach to identifying specific editing sites altered by ADAR3 expression in the glioblastoma transcriptome has not been performed. However, using the same RNA-seq datasets generated for the differential gene expression analysis in this study, we have been able to identify a list of nearly 400 editing sites impacted by ADAR3 expression^67^. Interestingly, many of those sites reside within 3′ untranslated regions, which if unedited, could potentially engage dsRNA sensors. While this list of cellular RNAs supports the possibility that ADAR3-mediated editing repression might contribute to NF-κB activation, determining individual transcripts or groups of transcripts that could engage dsRNA sensors represents a significant challenge. Therefore, future studies should directly test whether specific dsRNA sensors are required for the increased p65 phosphorylation, downstream gene expression changes and temozolomide resistance of ADAR3-expressing U87 cells. In addition, future analysis of these same molecular and cellular consequences in cells expressing ADAR3 mutants that alter RNA binding and/or editing repression is important to gain insight into the molecular mechanism by which ADAR3 expression impacts the glioblastoma transcriptome.

Transcriptomic studies suggest a negative correlation between ADAR3 mRNA expression and grade of glioblastoma malignancy^26,68^. However, it has been previously demonstrated that expression of ADAR3 protein does not correlate with the RNA level^25^, which limits the accuracy of predictive/prognostic analysis using TCGA and other large-scale datasets. Our current understanding of ADAR3 protein expression is restricted to a small number of cell lines and patient samples, hence a more comprehensive understanding of ADAR3 at the protein level in a larger patient sample dataset is needed. Furthermore, as our data suggests ADAR3-expressing cells have increased NF-κB activation, these data should be concordantly assessed in patient samples. As the literature regarding ADAR3 expression in glioblastoma patients is conflicting^8^, the results herein represent important steps towards determining whether ADAR3 could act as a potential driver of glioblastoma. Malignant glioma is thought to originate from mutations in neural stem cells and oligodendrocyte precursor cells (OPCs)^69^, and proliferation of these cell types is affected by proinflammatory cytokines such as TNF-α^70^. In our RNA-seq analysis, we observed that ADAR3-expressing U87 cells were significantly enriched for genes regulated by NF-κB in response to TNF-α^71^. More specifically, NF-κB regulated cytokines, such as IL-6, were upregulated in ADAR3-expressing U87 cells compared to control cells. IL-6 plays a vital role in the invasiveness of glioma, and immunohistochemical studies of patient-derived glioma tissues showed that IL-6 expression increased with the grade of glioma^72^. Therefore, ADAR3-mediated regulation of inflammatory response genes through NF-κB activation might be crucial in glioblastoma progression. However, our investigation was limited to glioblastoma cell culture where we increased ADAR3 expression via retroviral transduction. It would be beneficial to further explore the ADAR3-mediated gene regulatory functions in primary glioblastoma cells with elevated ADAR3 and corresponding CRISPR-generated ADAR3 knockout cells. One major challenge in glioblastoma treatment is cellular heterogeneity^73^. Cells such as microglia and tumor-associated macrophages secrete cytokines, including TNF-α and IL-6, within the tumor microenvironment to further modulate NF-κB signaling^74^. Therefore, future studies should investigate the impact of ADAR3-mediated NF-κB activation in response to stimuli or therapy in vivo using coculture and/or xenograft models. A better understanding of these pathways may be beneficial in developing effective immunotherapies against glioma.

While our studies focused on ADAR3 expression in glioblastoma cells, it is possible that ADAR3 might also be involved in the regulation of NF-κB signaling in normal functioning and pathogenesis in the central nervous system. ADAR3 is unique amongst the mammalian ADAR family members due to the exclusive expression of ADAR3 within brain tissues^21,75^, with the highest *ADAR3* expression detected in the hippocampus, amygdala, thalamus, and olfactory regions^21^. Impaired cognitive function, including increased anxiety and defects in hippocampus-dependent memory formation, has been previously reported in ADAR3 knockout mice^76^; however, the molecular mechanism by which loss of ADAR3 affects these behaviors was not uncovered. In the healthy brain, TNF-α signaling is involved in neurodevelopmental processes, synaptic plasticity^77^, learning, and memory^78^. TNF-α signaling also regulates neuroinflammatory responses associated with various neuronal disorders^79^. Similarly, loss of neuronal NF-κB signaling in the mature nervous system impairs learning and memory due to misregulation of axonal and dendritic growth and synaptic signaling^80^. Future studies in normal brain and neurodegenerative disorders should examine whether ADAR3 might be involved in TNF-α signaling and NF-κB activation required for proper nervous system development and function.

## Methods

### Cell culture and transfection

U87-MG (referred as U87 in the text), U118, and HEK293T Epstein-Barr nuclear antigen cells were grown in Dulbecco's modified Eagle's medium (DMEM) (Mediatech) supplemented with 10% fetal bovine serum (Sigma), 100 μg/mL penicillin, and 100 units/mL streptomycin (Mediatech). As previously described in Ref.^24^, the retroviral particles carrying neomycin/G418 resistant plasmid with no protein or 3X-FLAG ADAR3 were generated in HEK293T Epstein-Barr nuclear antigen cells. Then U87 and U118 cells were infected with the retroviral particles to generate control and ADAR3-expressing cells. After selection, U87 and U118 cells were maintained in 0.2 and 0.3 mg/mL G418 (neomycin), respectively. Expression of 3X-FLAG ADAR3 was confirmed by western blot using anti-ADAR3 antibody^67^. The Universal Mycoplasma Detection Kit (ATCC) was routinely used to verify that cell lines were free from mycoplasma contamination.

### RNA isolation and qRT-PCR

Total RNA was isolated using TRIzol (Invitrogen) and further purified by treatment with TURBO DNase (Ambion) followed by the RNeasy Extraction kit (Qiagen) and stored at − 80 °C. RNA concentrations and contamination with organic and protein components were determined with a Nanodrop (Fisher Scientific). For qRT-PCR, 2 ug of DNase-treated RNA was subjected to cDNA synthesis using Superscript III (Invitrogen) with random hexamers (Fisher Scientific) and oligo dT (Fisher Scientific) primers (Supplementary Table 2). After reverse transcription, 20 μL of water was added to the cDNA. Gene expression was determined using SybrFast Master Mix (KAPA) and gene-specific primers (Supplementary Table 2) on a Thermofisher Quantstudio 3 instrument. qRT-PCR primers spanned at least one exon boundary to prevent inappropriate detection of cross-reactivity from amplified genomic DNA products. The quality of the qRT-PCR products was assessed using melting curve analysis. For each gene analyzed, a standard curve of eight to ten samples of ten-fold serial dilutions of the amplicon were used to generate a standard curve of cycle threshold versus the relative concentration of amplicon. Standard curves were plotted on a logarithmic scale in relation to concentration and fit with a linear line. The fit of the lines (r^2^) ranged from 0.96 to 1, and all data points fell within the standard curve. Each cDNA measurement was performed in triplicate, and each experiment was performed in three biological replicates.

### Library preparation sequencing of polyadenylated RNA

Libraries were created from RNA isolated from three independent biological replicates of U87 cells expressing an empty vector and U87 cells expressing 3XFLAG-tagged ADAR3. PolyA + beads (Invitrogen) were used to select for mRNA, and libraries were generated using the KAPA Strand-Specific RNA Library Kit according to the manufacturer's instructions. Sequencing was performed by the Indiana University Center for Medical Genomics on an Illumina NextSeq500 instrument. In brief, 70–100 million, 75 bp paired-end, RNA-Seq reads were trimmed using fastp (version 0.20.1) with parameters “-l 17 -detect_adapter_for_pe -g -p”^81^. The resulting reads were mapped against GRCh38 using STAR (version 2.7.9a) with parameters “-outFilterMultimapNmax 25 -alignSJoverhangMin 8 -alignSJDBoverhangMin 1 -outFilterMismatchNmax 999 -outFilterMismatchNoverReadLmax 0.106 -alignIntronMin 20 -alignIntronMax 1,000,000 -alignMatesGapMax 1,000,000 -outSAMunmapped Within -outSAMtype BAM SortedByCoordinate”^82^. Read counts for each gene were created using featureCounts from the Subread package (version 2.0.2) with the parameters "-O -M -primary -p -countReadPairs -largestOverlap -B" and ENSEMBL release 103 as the annotation^83,84^. Differential expression analysis was performed using the DESeq2 package (version 1.30.1) in R/Bioconductor (R version 4.0.4)^85^.

### Gene set enrichment analysis

Genes that exhibited significant differential expression (p < 0.05, log_2_fold change >|0.5|) in ADAR3-expressing U87 cells compared to control cells (Supplementary Table 1) were selected for gene set enrichment analysis (GSEA) using the Molecular Signatures Database (V7.4)^43,44^. Gene set enrichment analysis for the differentially expressed genes was carried out by computing overlaps with hallmark gene sets (H) and setting the false discovery rate as q-value < 0.05. Similarly, GSEA analysis was performed on 10 random groups of 641 genes expressed in U87 cells. These random genes were selected from the unsorted list of genes with reads (> 1) in our ADAR3-expressing U87 RNA-seq dataset.

### Western analysis in U87 and U118 cells

U87 and U118 cells were plated at a density of 2 × 10^5^ cells/mL. For only the detection of phosphorylated IκB-α, cells were incubated with 5 µM MG132 (Cayman Chemical, 10012628) for 4 h before collection. After 24 h, the media was removed and washed with ice-cold 1X PBS. The cells were trypsinized and centrifuged at 1200*g* for 5 min. The cell pellet was washed with cold 1X PBS and resuspended in lysis buffer (2% SDS, 50 mM Tris–HCl, 10% Glycerol) with protease inhibitor (Roche) and kept on ice. The cells were sonicated and centrifuged at 15,000 rpm for 10 min. The Bradford assay was performed to determine the protein concentration of the supernatant. DTT (0.1 M) and Bromophenol blue (0.1%) were added, and lysates were boiled for 5 min. Equivalent amounts of protein lysates were subjected to SDS-PAGE and western blotting using antibodies against Phospho-S356-NF-κB p65 (Cell Signaling, 3033), NF-κB p65 (Cell Signaling, 8242), NF-κB p52/p100 (Santa Cruz, 7386), IκB-α (Cell Signaling, 4814), Phospho-S32/36-IκB-α (Cell Signaling, 9246) and β-Actin (Cell Signaling, 8457S). To minimize inaccuracies from stripping and reprobing immunoblots, whenever possible, blots were cut to allow for simultaneous probing of loading controls and proteins of interest. For experiments where multiple proteins were analyzed on one immunoblot, primary antibodies were added after the blots were cut. For reprobing the membrane was washed in 1X TBS twice for 5 min each and incubated with mild stripping buffer (0.2 mM Glycine, 0.1% SDS, 1% Tween 20, pH 2.2) at room temperature with agitation for 20 min. The membrane was then washed three times with 1X TBS for 5 min before proceeding to blocking and reprobing. Protein bands were visualized using enhanced chemiluminescent detection reagents (Thermo Scientific). The images in the dynamic range without saturation were acquired using Image Lab software (version 6.1.0 build 7) in the BIO-RAD ChemiDoc MP imaging system. The band intensity of phospho-p65 and p65 was quantified using ImageJ software (version 1.53 k) and normalized by the band intensity of β-actin from the corresponding blot. Full length immunoblot images of the figures in the manuscript are included in the supplementary information file.

### TNF-α treatment analysis

Cells were cultured in 6-well plates at a cell density of 5 × 10^5^ cells/well for 24 h in DMEM with 10% FBS, 100 μg/mL penicillin, and 100 units/mL streptomycin. Cells were treated with 0 or 10 ng/mL of TNF-α and incubated at 37 °C for 0, 1, or 2 h. After incubation, cells were washed with ice-cold 1X PBS and collected. The cells were subjected to western analysis, RNA isolation and qRT-PCR as mentioned above.

### Nuclear-cytoplasmic fractionation

U87 cells expressing 3X FLAG-ADAR3 were washed with 1X PBS and trypsinized. Cells were collected by centrifugation at 1000×*g* for 3 min at 4 °C. After washing twice with 1X PBS, the cell pellet was resuspended in 500 µL lysis buffer (10 mM Tris, pH 8.4, 140 mM NaCl, 1.5 mM MgCl_2_, 0.5% NP-40, 1 mM DTT) and 50 µL of cell suspension was transferred to a new tube (whole-cell sample). Nuclei were pelleted by centrifugation at 1000×*g* for 5 min at 4 °C. The supernatant (cytoplasmic fraction) was transferred to a new tube. Nuclei were resuspended in 500 µL lysis buffer and then 50 µL of detergent solution (3.3% [wt/wt]) sodium deoxycholate and 6.6% [vol/vol]) Tween 20) was added, and the tube was vortexed slowly. After incubation on ice for 5 min, nuclei were pelleted by centrifugation at 1000×*g* for 5 min at 4 °C. The supernatant was added to the cytoplasmic fraction collected in the previous step. The nuclei were suspended in an equal volume of lysis buffer as cytoplasmic fraction. Equivalent amounts of samples were subjected to SDS-PAGE and transferred western blotting with antibodies to NF-κB p65 (Cell Signaling, 8242), Tubulin (Sigma, T-9026), and Histone H3 (Cell Signaling, 4620S).

### Poly I:C infection and qRT-PCR

Cells were seeded in a 10 cm plate at a concentration of 1 × 10^5^ cells/mL in Dulbecco's modified Eagle's medium (DMEM) (Mediatech) supplemented with 10% fetal bovine serum (FBS) (Sigma). After 24 h, cells were transfected with 500 ng/mL of poly I:C (Sigma) using FuGENE HD transfection reagent (Promega). Cells were incubated with poly I:C at 37 °C for 0, 16, and 24 h. Total RNA isolation and qRT-PCR were performed as mentioned above.

### Luciferase assay

Cells were plated at a density of 2 × 10^4^ cells/well in 96-well plates and incubated at 37 °C. After 24 h, the cells were transfected with a reporter plasmid, which expresses firefly luciferase under the control of the NF-κB promoter (pNF-κB-Luc)^86^ and pRenilla-Luc, which constitutively expresses *Renilla* luciferase. The transfection used FuGENE HD (Promega) and was performed according to the manufacturer's instructions. After 24 h, Firefly and *Renilla* luciferase activity were quantified using a dual luciferase assay kit (Promega) according to the manufacturer's instructions.

### Cell viability assay

For temozolomide treatment, control and ADAR3-expressing U87 and U118 cells were counted and plated at 2000 cells/well density in 96 well plates in triplicates and incubated at 37 °C for 24 h. Cells were then treated with DMSO or 200–500 μM temozolomide (dissolved in DMSO) for 0, 24 or 48 h. Cytotoxicity was monitored by MTT (3-(4,5-dimethylthiazol-2-yl)-2,5-diphenyltetrazolium bromide) tetrazolium) assay. The MTT stock solution was made at a 5 mg/mL concentration in 1X PBS and was diluted to 1 mg/mL in growth medium immediately before addition to cells. After 50 μL of MTT solution was added, cells were incubated at 37 °C. After 4 h, the MTT-containing medium was replaced with 150 μL DMSO and absorbance was measured at 600 nm using a microplate reader. The percentage of cells surviving the treatment was calculated as follows: % Cell viability = [A_600_ (t) − A_600_ (0)]_TMZ_**/**[A_600_ (t) − A_600_ (0)]_DMSO_.

For examining resistance to ionizing radiation, ADAR3-expressing U87 cells and control cells were exposed to either 0 Gy or 5 Gy of ionizing radiation. After 24 h, 1000 cells from each cell line were seeded in triplicate onto a 96-well tissue culture plate and incubated at 37 °C for 0, 48 and 72 h. MTT assay was performed as described above. The percentage of cells surviving the 5 Gy of ionization radiation in each cell line was calculated as follows: % Cell viability = [A_600_ (t) − A_600_ (0)]_5 Gy_**/**[A_600_ (t) − A_600_ (0)]_0 Gy_ × 100.

For examining the effect of BMS345541 (Sigma, 401480) treatment on cell viability, control and ADAR3-expressing U87 cells were counted and plated at 2000 cells/well density in 96 well plates in triplicates and incubated at 37 °C for 24 h. Cells were then treated with DMSO or 5 µM BMS345541 (dissolved in DMSO) for 0, 24 or 48 h and an MTT assay was performed as described above.

### Statistical analysis

All data was plotted and analyzed using GraphPad Prism. The statistical test employed for each dataset is mentioned in the corresponding figure legend.

## Supplementary Information

Supplementary Table S1.

Supplementary Table S2.

Supplementary Figure S1.

Supplementary Figure S2.

Supplementary Figure S3.

Supplementary Figure S4.

Supplementary Figure S5.

Supplementary Information.

## Data Availability

High-throughput sequencing data can be accessed at the Gene Expression Omnibus (GSE198547).
