# Supplementary material for: ADAR3 activates NF-κB signaling and promotes glioblastoma cell resistance to temozolomide
Source: Sci Rep. 2022 Aug 3;12:13362. doi: 10.1038/s41598-022-17559-4 (PMC9349284; doi:10.1038/s41598-022-17559-4)
Supplement: Supplementary file 3 — Supplementary Figure S1. [file 41598_2022_17559_MOESM3_ESM.pdf]

**Figure S1**

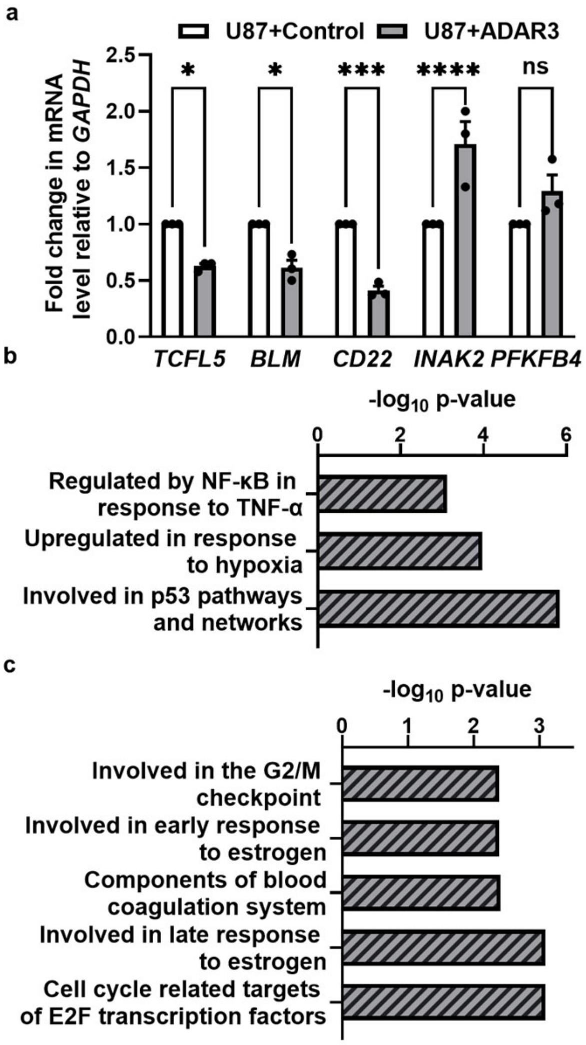


**Supplementary Figure S1.** Gene expression changes in U87 cells upon ADAR3 expression. (**a**) Expression of each indicated gene was determined relative to the expression of *GAPDH* determined by qRT-PCR in the same cell lines. The relative expression values were normalized to the control U87 cell line. The mean of three biological replicates is plotted with error bars representing standard error of the mean. Statistical significance was determined using ordinary two-way ANOVA with Sidak's multiple comparisons test. *$p \leq 0.05$, ***$p \leq 0.0005$, ****$p < 0.0001$ (**b, c**) Significantly enriched pathways of up (**b**) and downregulated (**c**) genes are listed in ascending order of the -log10 p-value provided by the Gene Set Enrichment Analysis computational method.
