# Supplementary material for: ADAR3 activates NF-κB signaling and promotes glioblastoma cell resistance to temozolomide
Source: Sci Rep. 2022 Aug 3;12:13362. doi: 10.1038/s41598-022-17559-4 (PMC9349284; doi:10.1038/s41598-022-17559-4)
Supplement: Supplementary file 4 — Supplementary Figure S2. [file 41598_2022_17559_MOESM4_ESM.pdf]

Figure S2

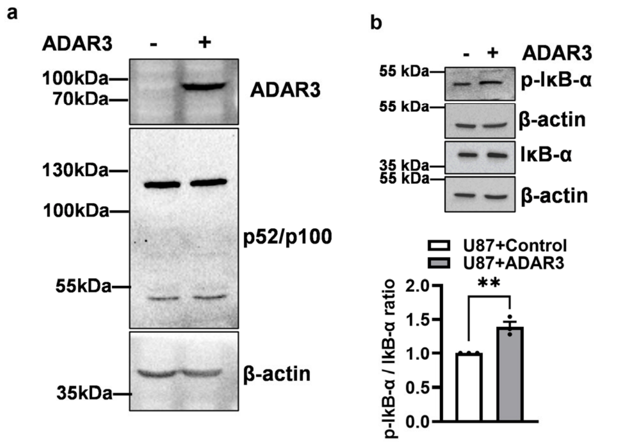

Supplementary Figure S2. ADAR3 expression promotes activation of the canonical NF-κB pathway. (**a**) Control (ADAR3 -) and ADAR3-expressing (ADAR3 +) U87 cells were lysed and subjected to immunoblotting with antibodies to ADAR3, NF-κB2 p52/ p100, and β-actin. Blot is a representative image (replicate 2) of three biological replicates and the uncropped images are included in the supplementary information file. (**b**) Control and ADAR3-expressing cells were treated with 5µM MG132 for 4 h, and cell lysates were collected and subjected to immunoblotting with antibodies to phosphorylated IκB-α (p-IκB-α), IκB-α and β-actin and quantified. Blot is a representative image (replicate 2) of three biological replicates and the uncropped blot images are included in supplementary information file. The ratio of p-IκB-α to total IκB-α relative to β-actin controls for each blot was determined in both ADAR3-expressing and control cells lines and normalized to the control cell line. Error bars represent the standard error of the mean (SEM) for three biological replicates. Statistical significance was determined using a two-tailed unpaired t-test. $^{**}p \leq 0.005$
