# Supplementary material for: ADAR3 activates NF-κB signaling and promotes glioblastoma cell resistance to temozolomide
Source: Sci Rep. 2022 Aug 3;12:13362. doi: 10.1038/s41598-022-17559-4 (PMC9349284; doi:10.1038/s41598-022-17559-4)
Supplement: Supplementary file 5 — Supplementary Figure S3. [file 41598_2022_17559_MOESM5_ESM.pdf]

**Figure S3**

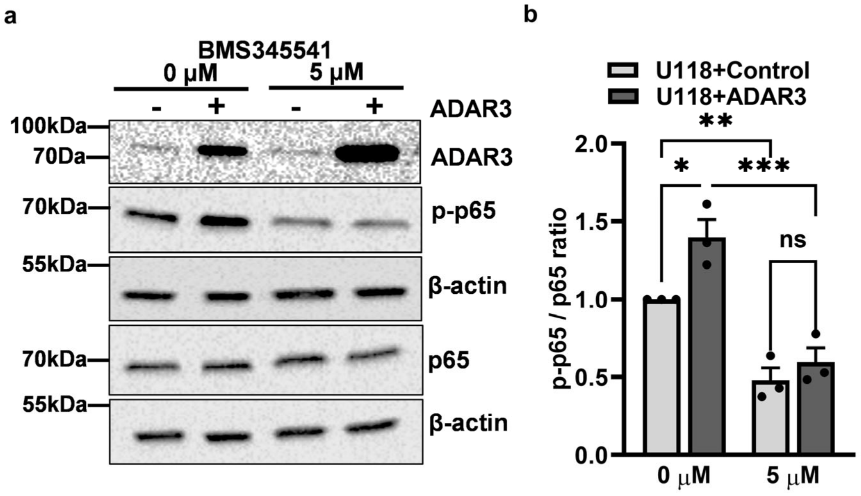


**Supplementary Figure S3. ADAR3 expression leads to increased p65 phosphorylation in U118 glioblastoma cells.** (**a**) Control and ADAR3-expressing U118 cells were treated with 0 (DMSO only) or 5 μM of BMS345541 for 24 h. An equivalent amount of cell lysates was subjected to quantitative immunoblotting with antibodies against p65, phosphorylated S356 p65 (p-p65), and β-actin. Blot is a representative image (replicate 1) of three biological replicates and the original images are included in supplementary information file. (**b**) The ratio of p-p65 to total p65 relative to β-actin was quantified and normalized to U118 control cells with 0 μM BMS345541 treatment. The mean of three biological replicates is plotted with error bars representing SEM. Statistical significance was determined using two-way ANOVA Tukey's multiple comparisons test. *p ≤ 0.05, **p ≤ 0.005 ***p ≤ 0.0005, ns indicates no significant difference.
