# Supplementary material for: ADAR3 activates NF-κB signaling and promotes glioblastoma cell resistance to temozolomide
Source: Sci Rep. 2022 Aug 3;12:13362. doi: 10.1038/s41598-022-17559-4 (PMC9349284; doi:10.1038/s41598-022-17559-4)
Supplement: Supplementary file 6 — Supplementary Figure S4. [file 41598_2022_17559_MOESM6_ESM.pdf]

Figure S4

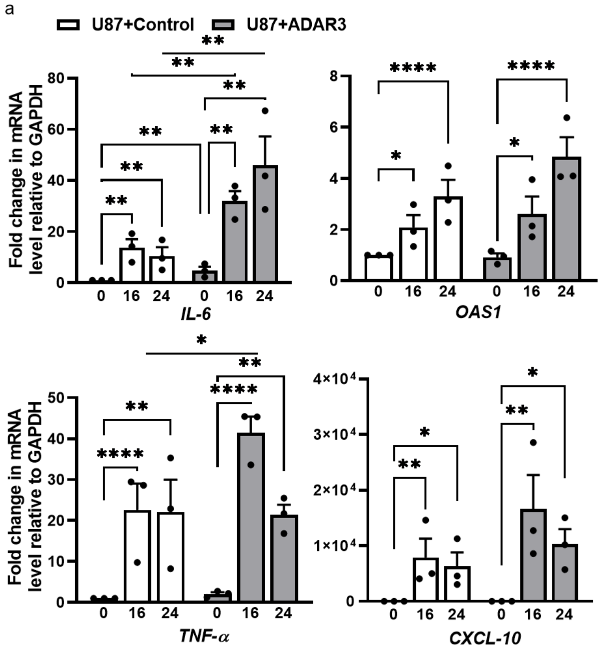

Supplementary Figure S4. ADAR3-expressing cells exhibit increased expression of NF-κB target genes in response to poly I:C treatment. Cells of the indicated genotypes were transfected with 500 ng/ml of Poly I:C for 0, 16, and 24 h. Expression of each indicated gene was determined relative to the expression of *GAPDH* determined by qRT-PCR in the same cell lines and was normalized to the expression of same gene relative to *GAPDH* in control cells at 0 h. The mean of three biological replicates is plotted with error bars representing SEM. Statistical significance was determined using two-way ANOVA Fisher's LSD test. *$p \leq 0.05$, **$p \leq 0.005$, ****$p < 0.0001$.
