# Supplementary material for: ADAR3 activates NF-κB signaling and promotes glioblastoma cell resistance to temozolomide
Source: Sci Rep. 2022 Aug 3;12:13362. doi: 10.1038/s41598-022-17559-4 (PMC9349284; doi:10.1038/s41598-022-17559-4)
Supplement: Supplementary file 7 — Supplementary Figure S5. [file 41598_2022_17559_MOESM7_ESM.pdf]

Figure S5

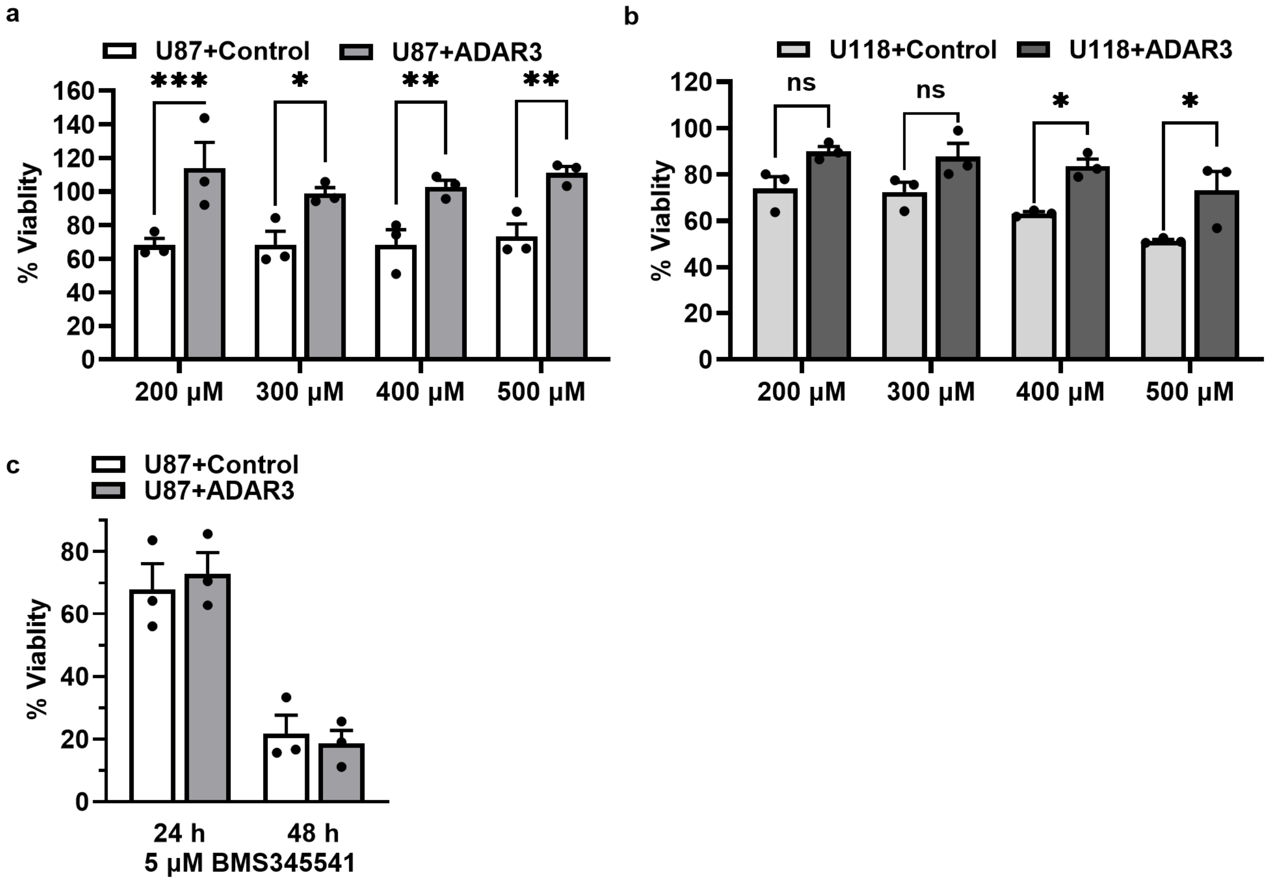

Supplementary Figure S5. **ADAR3 expression increases glioblastoma cell survival to temozolomide.** (**a, b**) Cell viability of ADAR3-expressing U87 (**a**) and U118 (**b**) cells compared to the respective control cells was determined using an MTT assay. Cells were treated with DMSO or the indicated concentration of temozolomide (dissolved in DMSO) for 24 h. The absorbance obtained at 0 h for each sample was subtracted from the 24 h readings, and live cells in the TMZ treated cells are represented as the percentage of corresponding DMSO treated cells. (% Cell viability = $[A_{600}(t) - A_{600}(0)]_{treatment} / [A_{600}(t) - A_{600}(0)]_{control}$). (**a, b**) The mean of three biological replicates is plotted with error bars representing SEM. Statistical significance was determined using two-way ANOVA Fisher's LSD test. $*p \leq 0.05$, $**p \leq 0.005$, $***p \leq 0.0005$. (**c**) Cell viability of control and ADAR3-expressing U87 cells treated with 5µM BMS345541 or DMSO for 24 and 48 h was determined using an MTT assay as described in (**a**).
