# Supplementary material for: ADAR3 activates NF-κB signaling and promotes glioblastoma cell resistance to temozolomide
Source: Sci Rep. 2022 Aug 3;12:13362. doi: 10.1038/s41598-022-17559-4 (PMC9349284; doi:10.1038/s41598-022-17559-4)
Supplement: Supplementary file 8 — Supplementary Information. [file 41598_2022_17559_MOESM8_ESM.pdf]

Supplementary Information

This file contains full length immunoblot images of figures in the manuscript grouped by the immunoblot in which the samples were run. The cropped images of blots used in the figures in manuscript are marked in red boxes.

Figure 2a

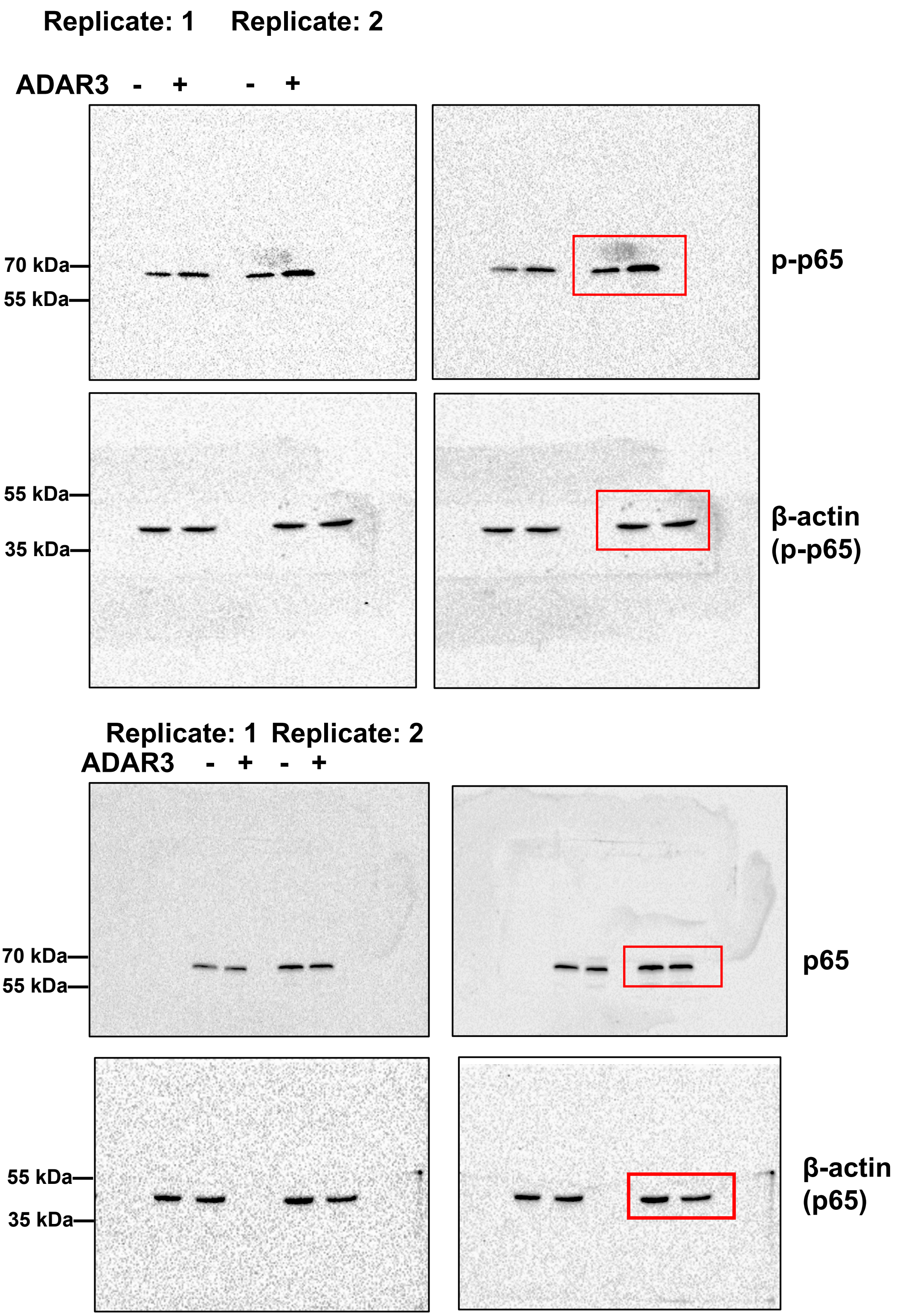

The entire blot images corresponding to Fig. 2a.The blots were cut at 55 kDa in order to develop using antibody to p65 or p-p65 and β-actin. Two exposures of each blot are shown.

Figure 2a

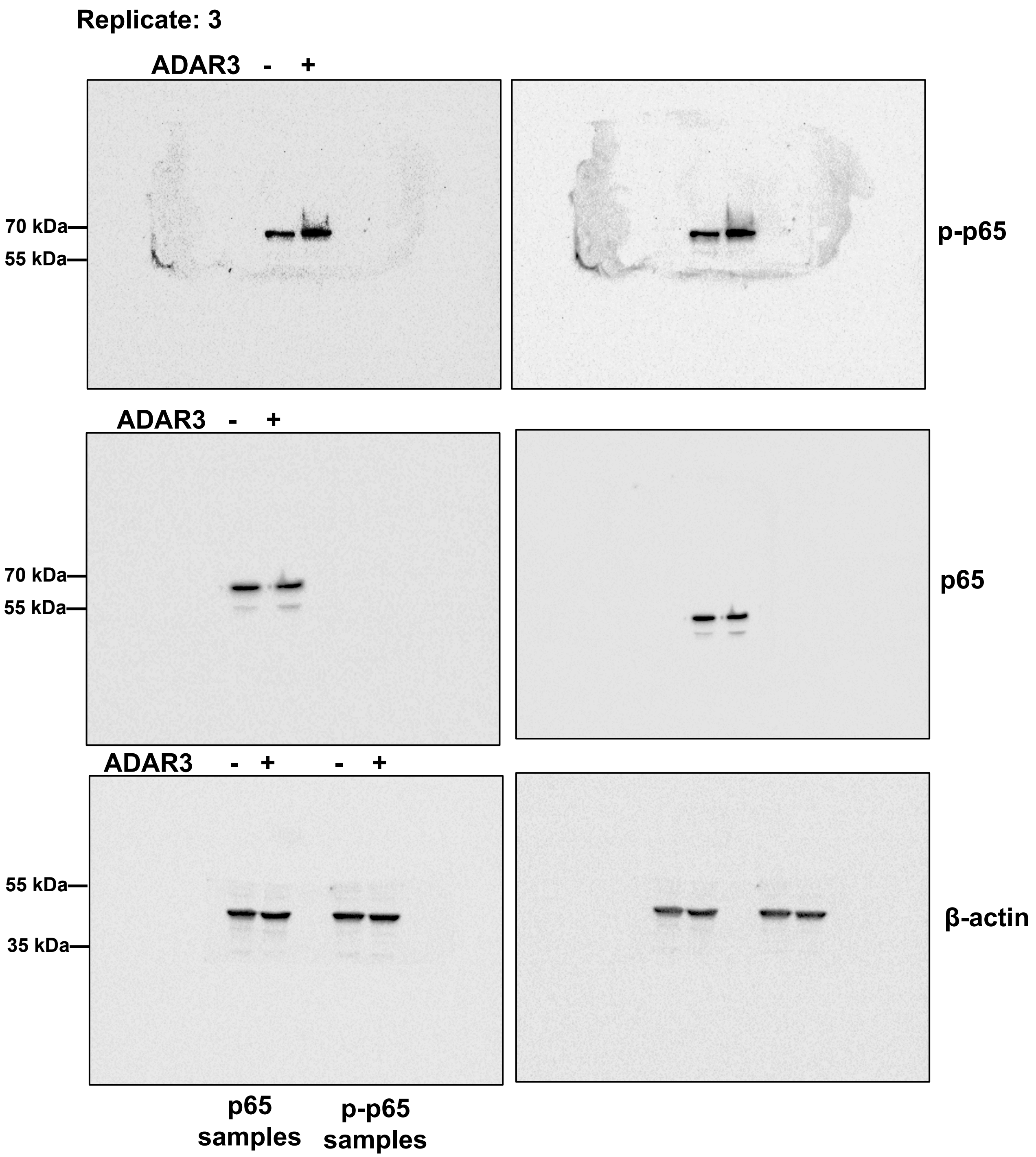

The entire blot images corresponding to Fig. 2a.The blots were cut at 55 kDa in order to develop using the p65 or p-p65 and β-actin. Two exposures of each blot are shown.

Figure 2a

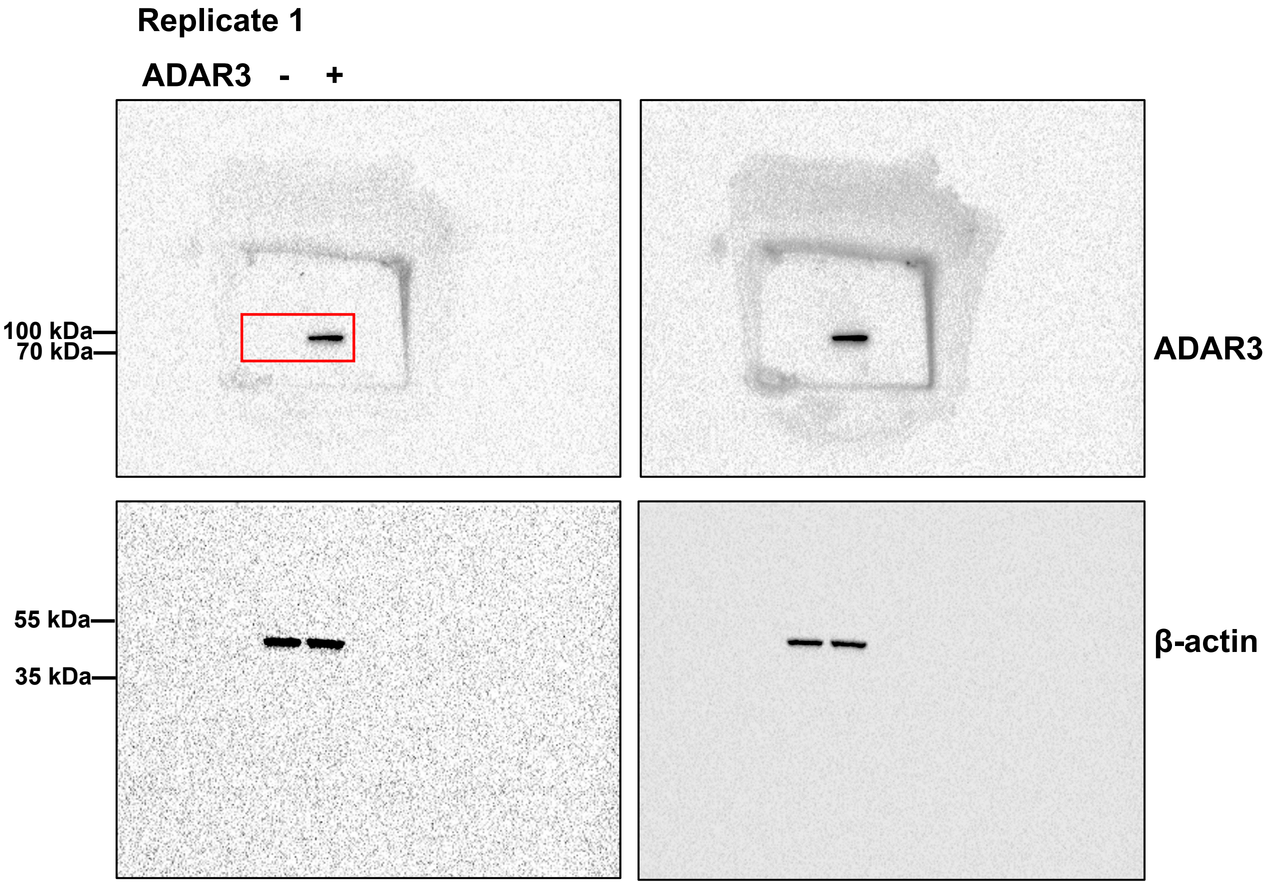

The entire blot images corresponding to Fig. 2a.The blots were cut at 55 kDa in order to develop using the ADAR3 and β-actin antibody. Two exposures of each blot are shown.

Figure 2c

Replicate 1

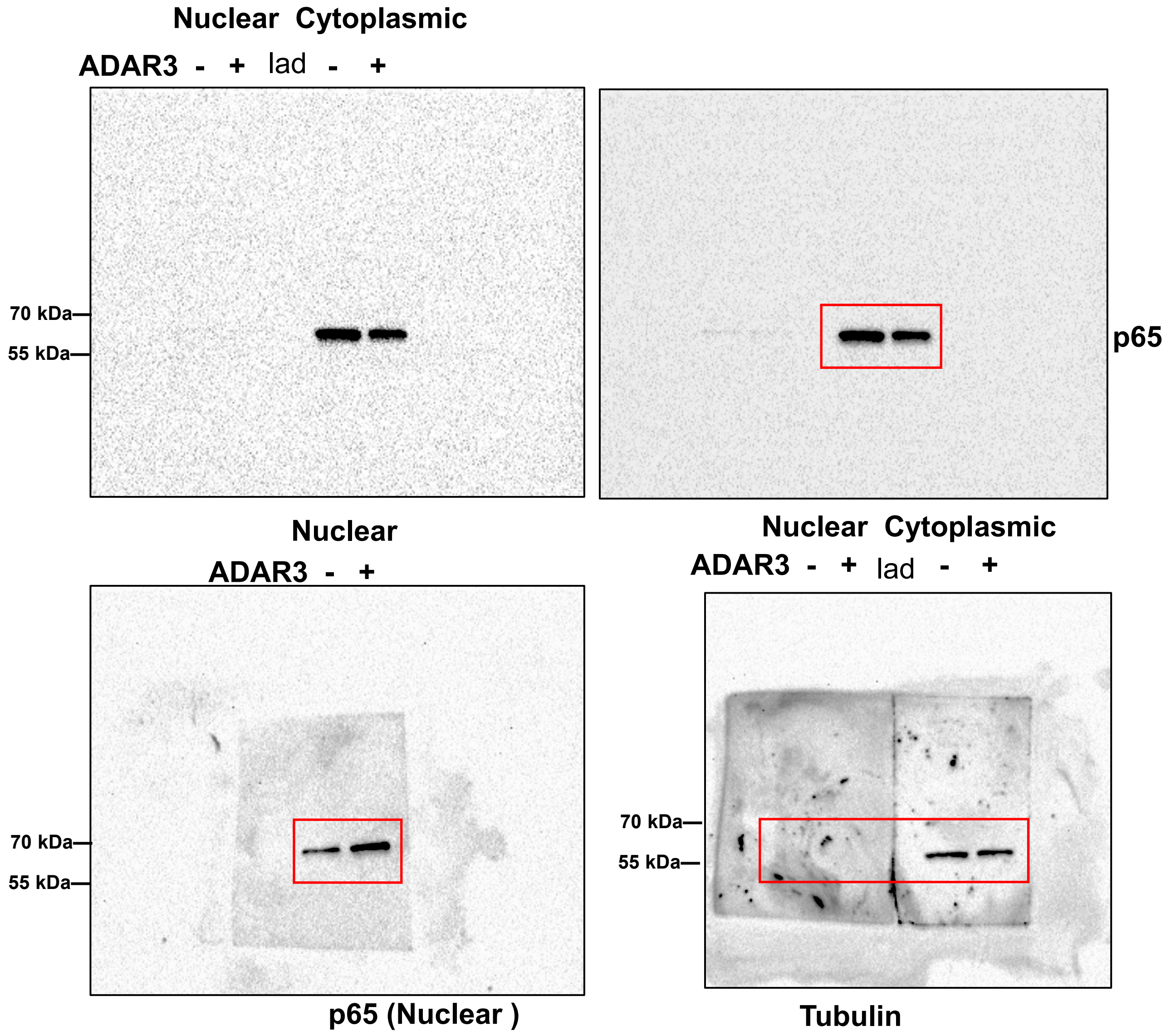

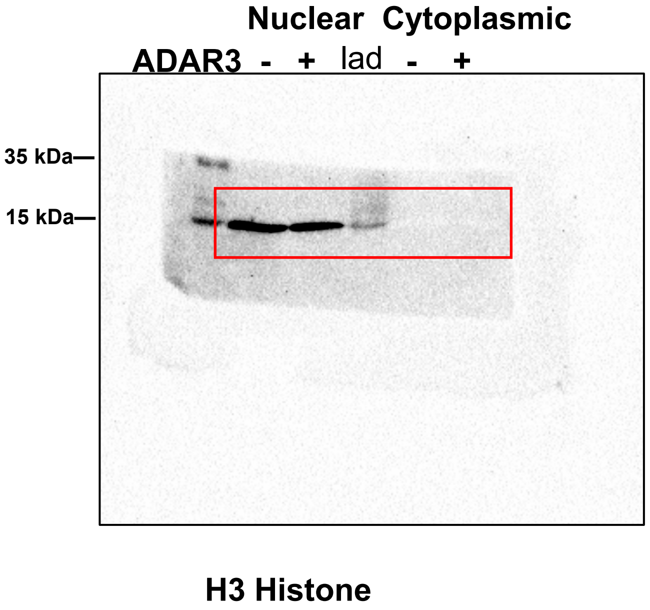

The entire immunoblots corresponding to Fig. 2c are shown. All the nuclear-cytoplasmic fractionation blots were first cropped at ~35 kDa to develop H3 histone and p65. Then, the upper region of the immunoblot was cropped at the lane with ladder (lad) and the nuclear fractions were developed for p65 separately. Then both membranes with nuclear and cytoplasmic fractions were stripped and reblotted for tubulin together.
Images from two exposures of cytoplasmic p65 blots are included.

Figure 2c

Replicate 2

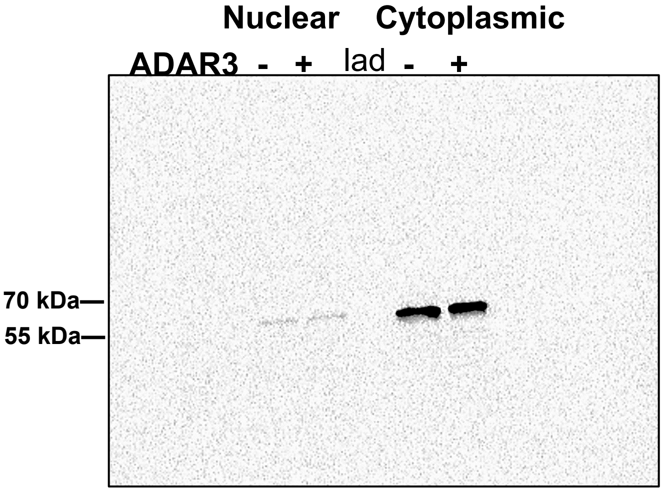

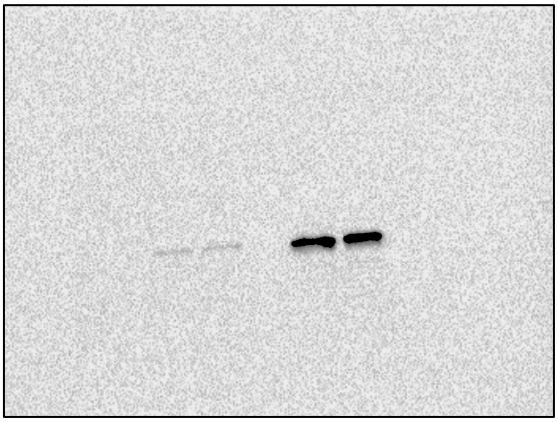

p65

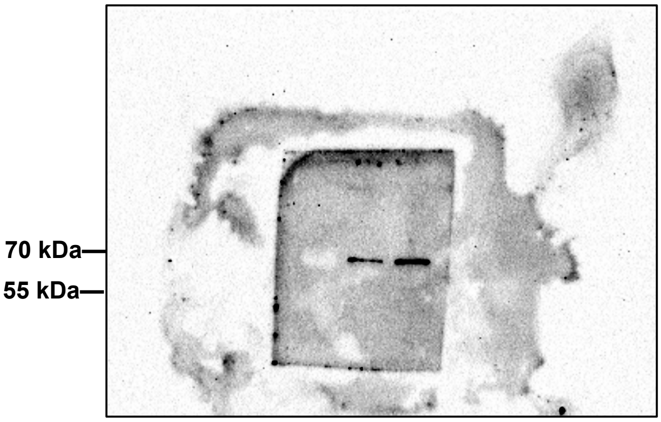

p65 (Nuclear)

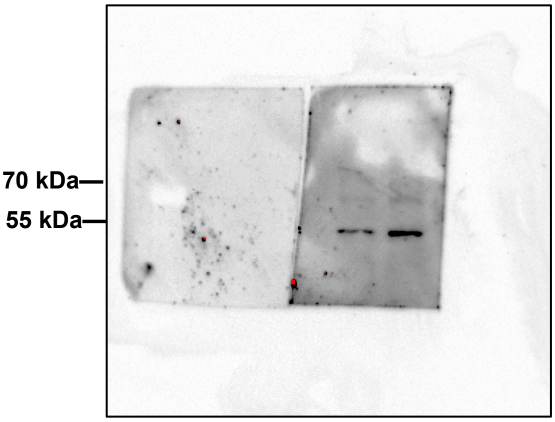

Tubulin

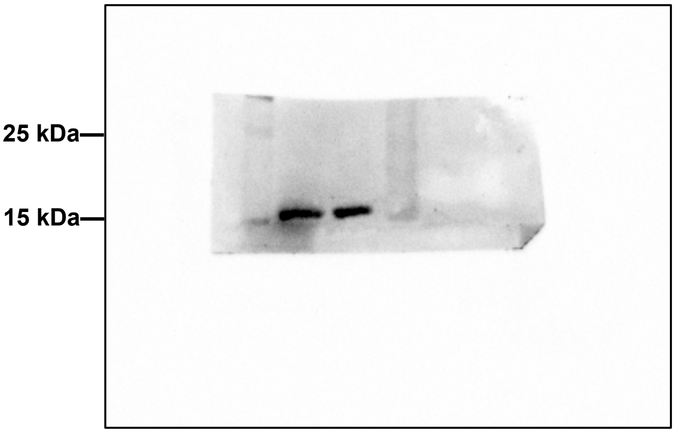

H3 Histone

Images of entire blots corresponding to Fig.2c are shown. The blots were analyzed the same as mentioned for Replicate 1. Images from two exposures of cytoplasmic p65 blots are included.

Replicate 3

Figure 2c

Nuclear Cytoplasmic
ADAR3 - + lad - +

70 kDa—
55 kDa—

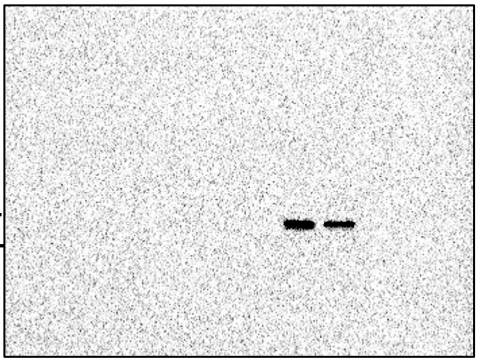

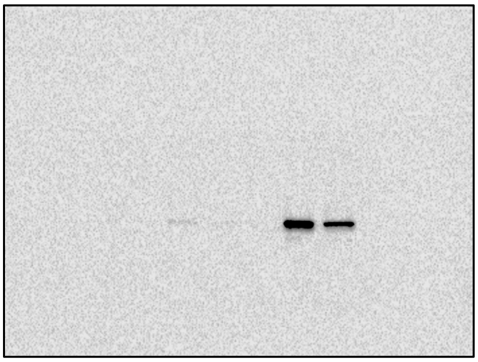

p65

Nuclear
ADAR3 - +

70 kDa—
55 kDa—

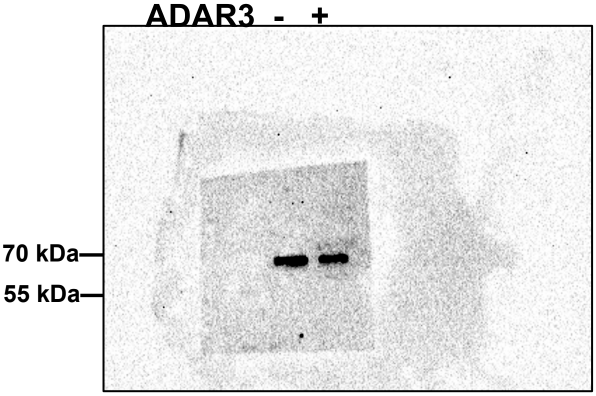

p65 (Nuclear)

Nuclear Cytoplasmic
ADAR3 - + lad - +

70 kDa—
55 kDa—

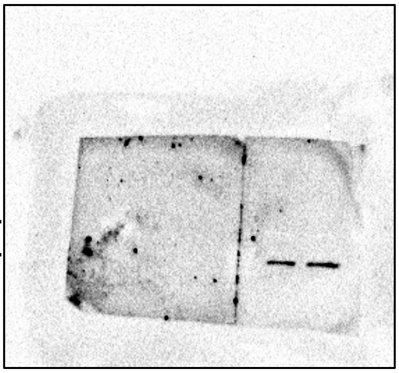

Tubulin

Nuclear Cytoplasmic
ADAR3 - + lad - +

35 kDa—
15 kDa—

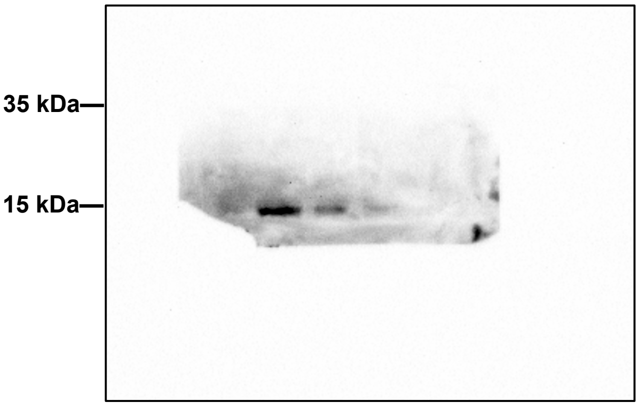

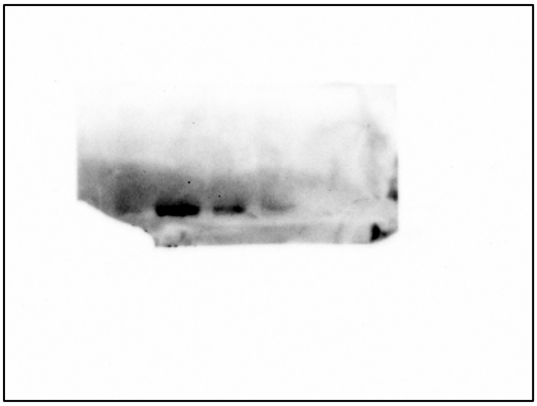

H3 Histone

Images of entire blots corresponding to Fig.2c are shown. The blots were analyzed the same as mentioned for Replicate 1.
Images from two exposures of cytoplasmic p65and Histone H3 blots are included.

Figure S2a

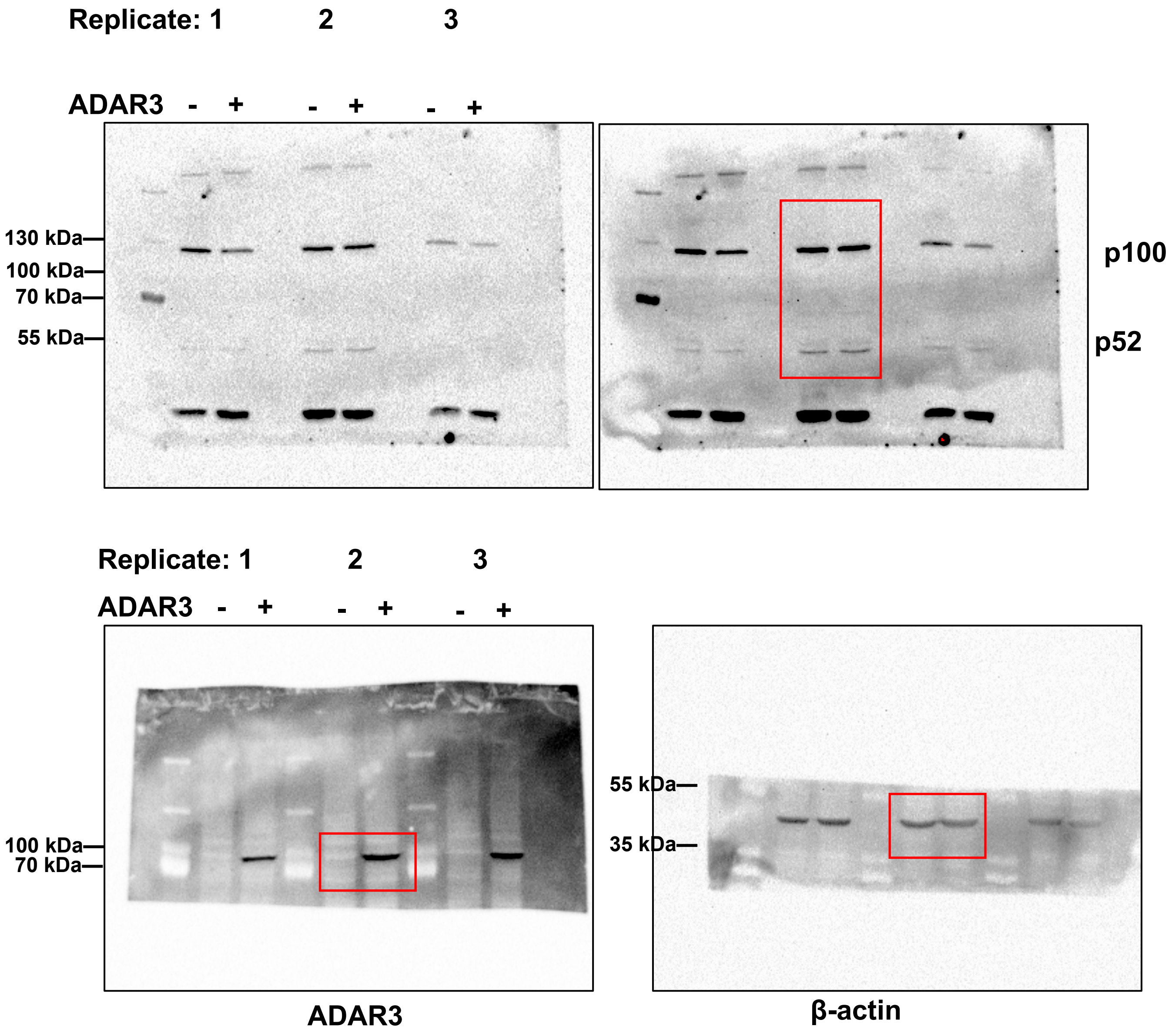

Images of entire blots corresponding to Fig. S2a are shown. The full-length blot was developed using p52/p100 antibody. The blot was then cut at 55kDa and reprobed for ADAR3 and β –actin.

Figure S2b

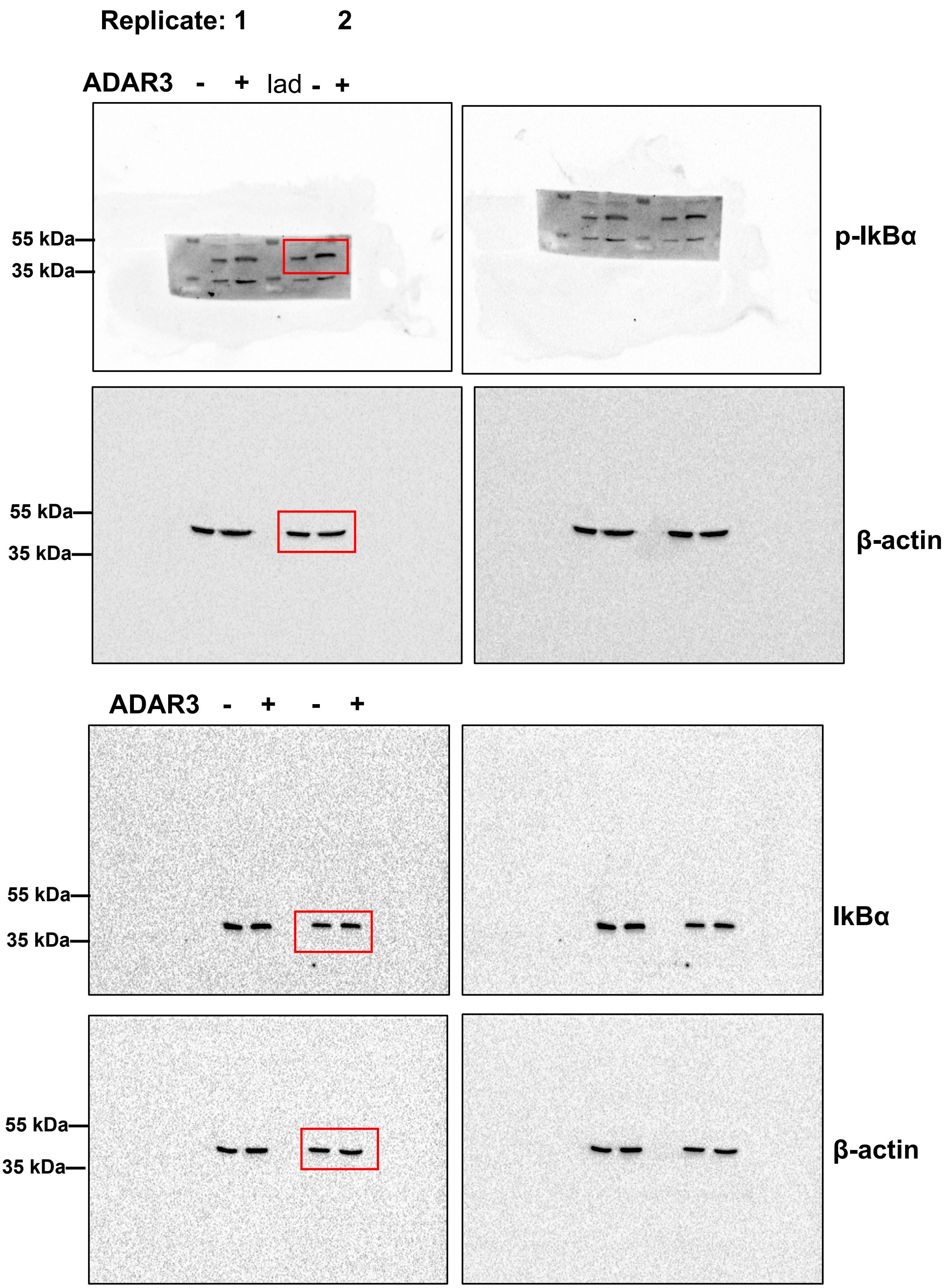

Images of entire blots corresponding to Fig. S2b are shown. Membrane was developed using IkBα and p-IkBα antibody and then reprobed with β-actin.

Figure S2b

Replicate 3

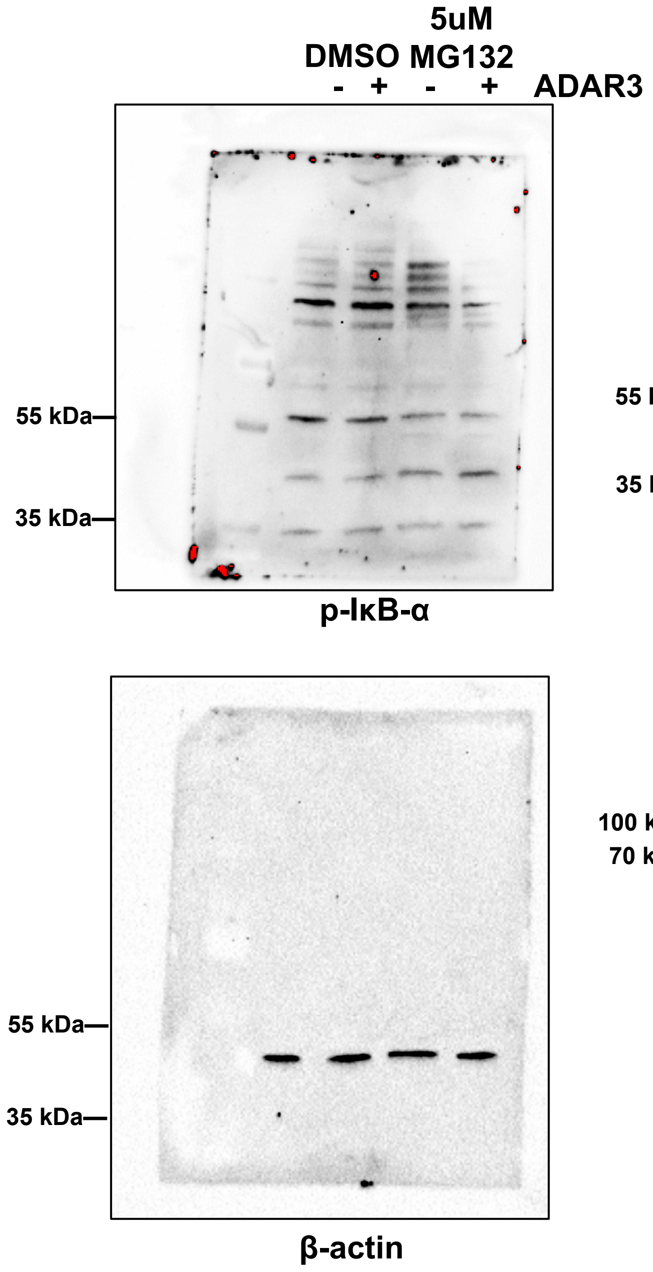

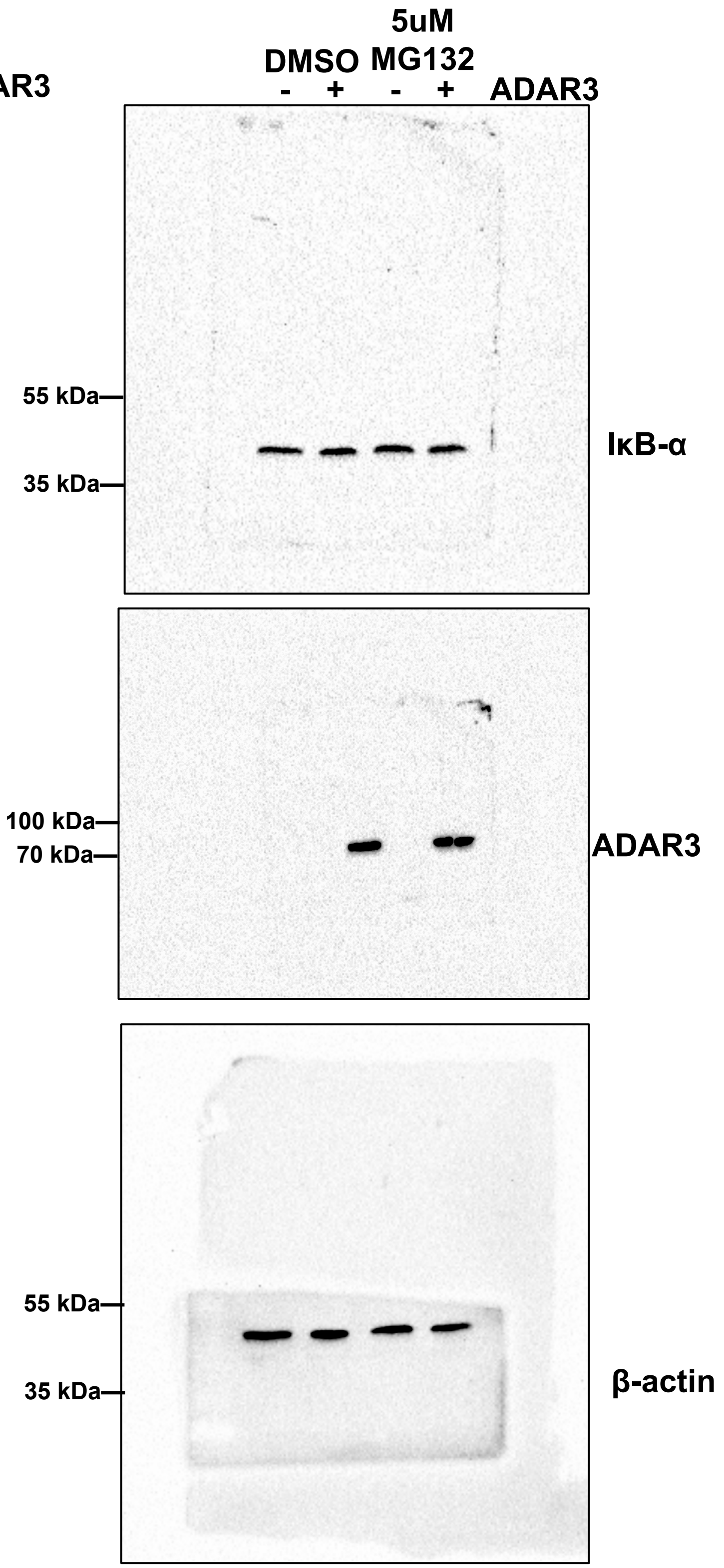

Images of entire blots corresponding to Fig. S2b are shown. Membrane was developed using IkBα and p-IkBα antibody, then reprobed with ADAR3 and β-actin antibody.

Figure 3c

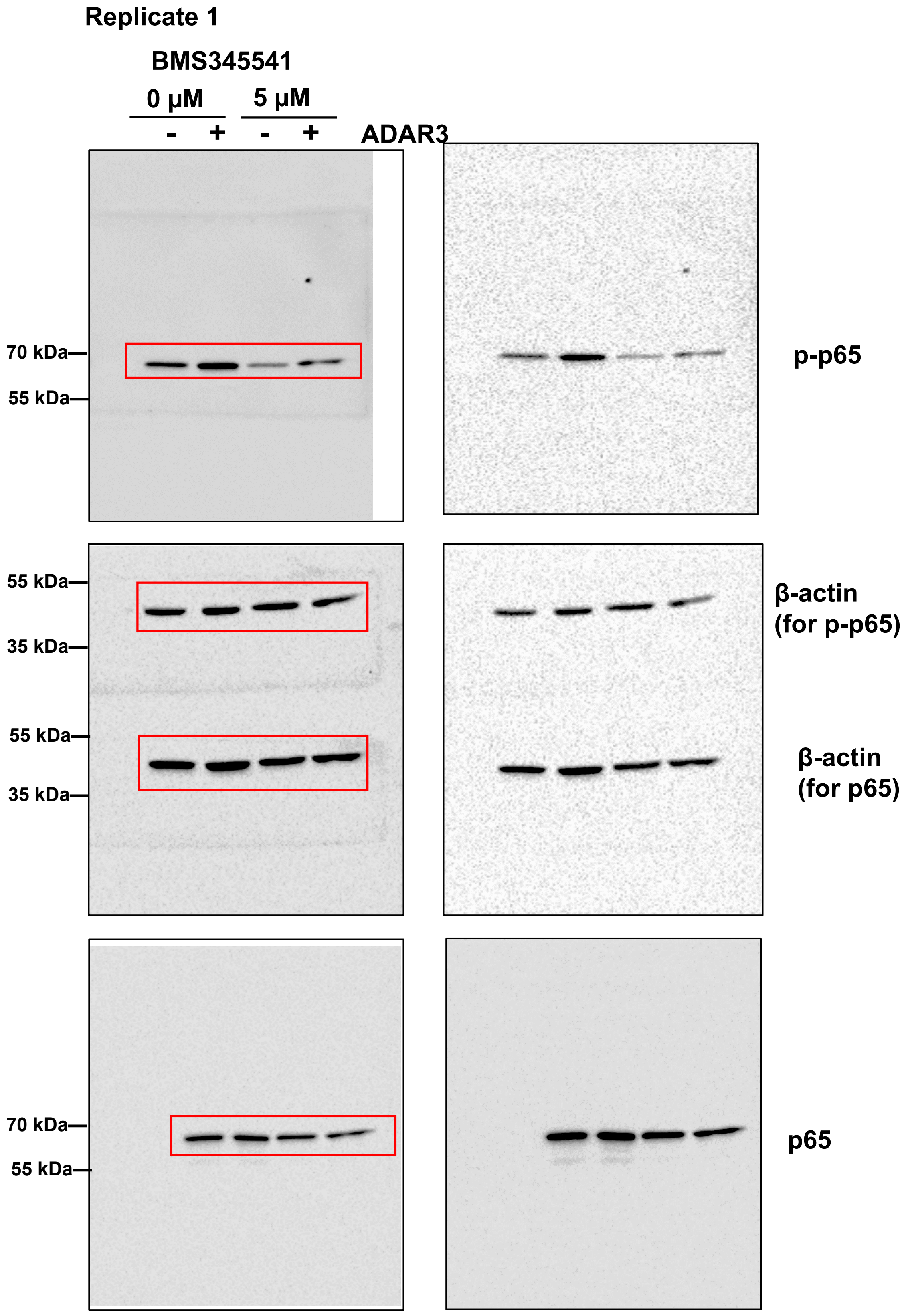

The entire blot images corresponding to Fig 3C are shown. The blots were cut at 55kDa to develop using p65 or p-p65 and β-actin antibodies. Two exposures of each blot are included. The β-actin blots corresponding to p65 (lower) and p-p65 (upper) were imaged together. The samples are loaded in the same order in both gels

Figure 3c

Replicate 2

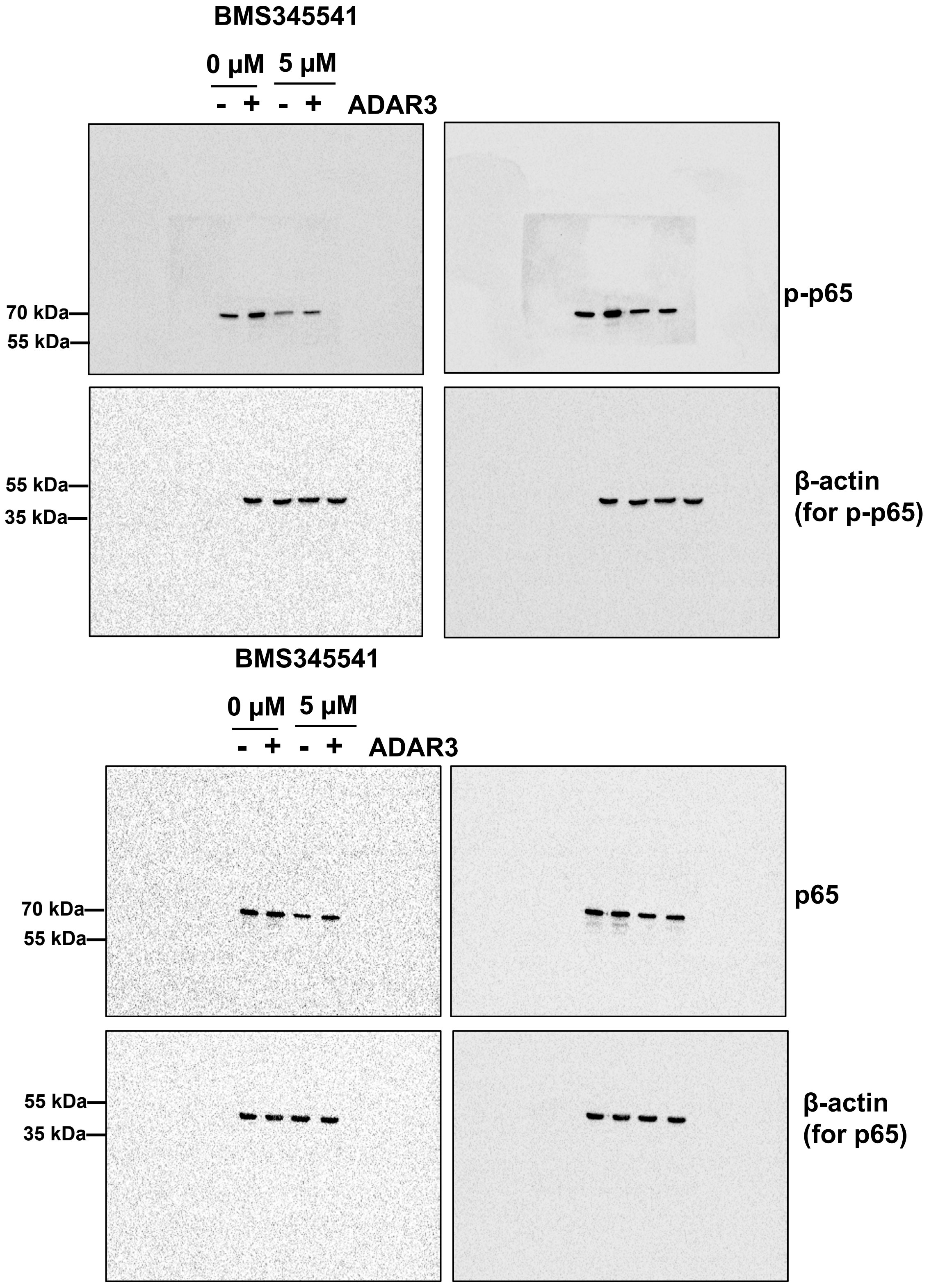

The entire blot images corresponding to Fig 3c. The blots were cut at 55 kDa in order to develop using antibody to p65 or p-p65 and β-actin. Two exposures of each blot are shown.

Figure 3c

Replicate 3

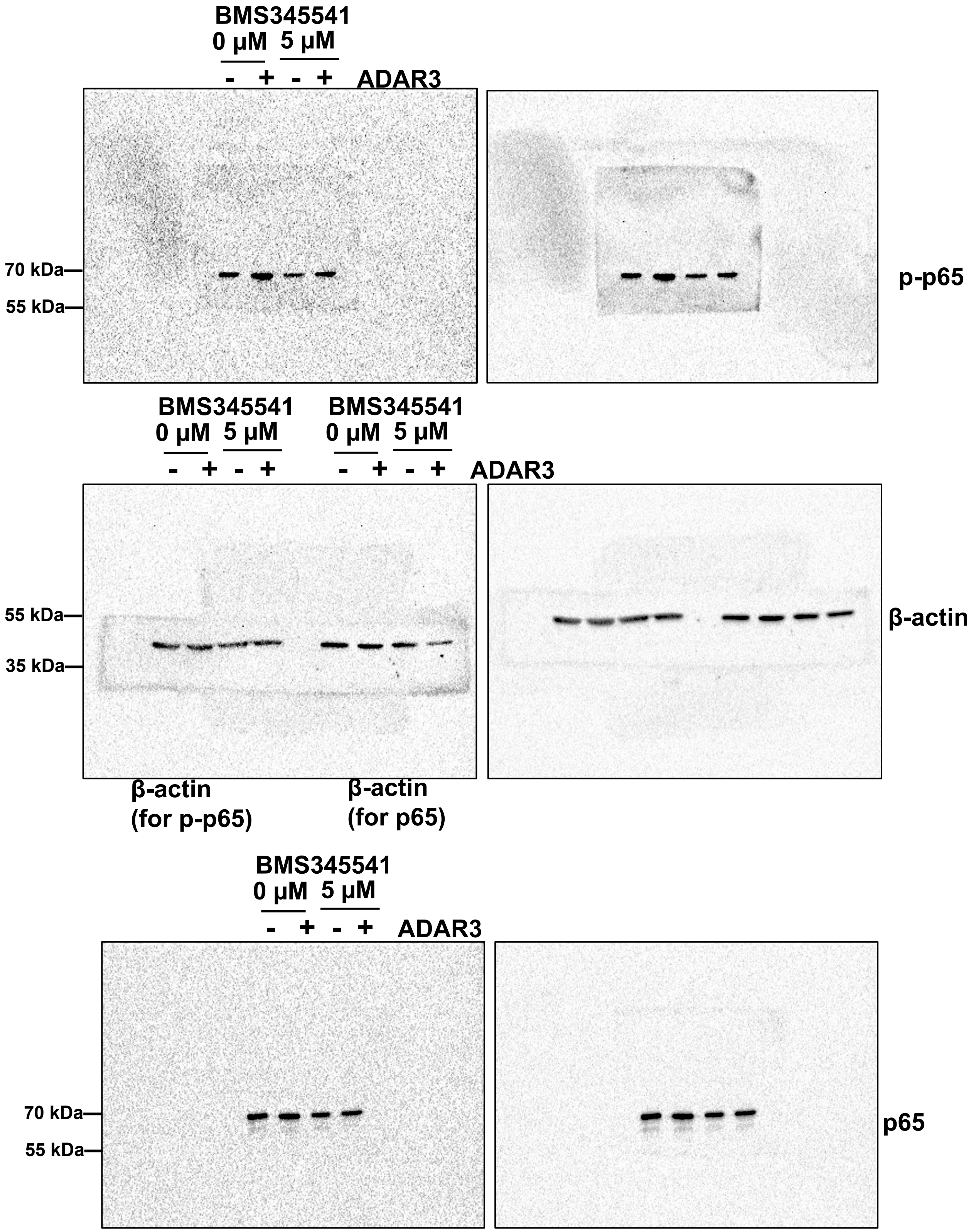

The entire blot images corresponding to Fig 3c. The blots were cut at 55 kDa in order to develop using antibody to p65 or p-p65 and β-actin. Two exposures of each blot are shown.

Figure 3c

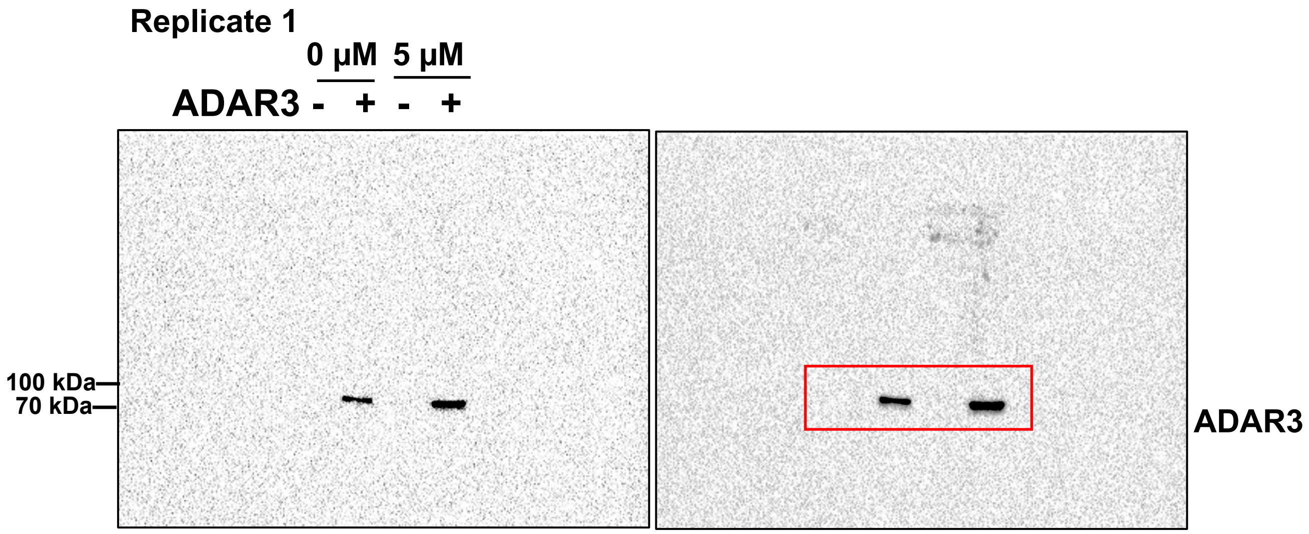

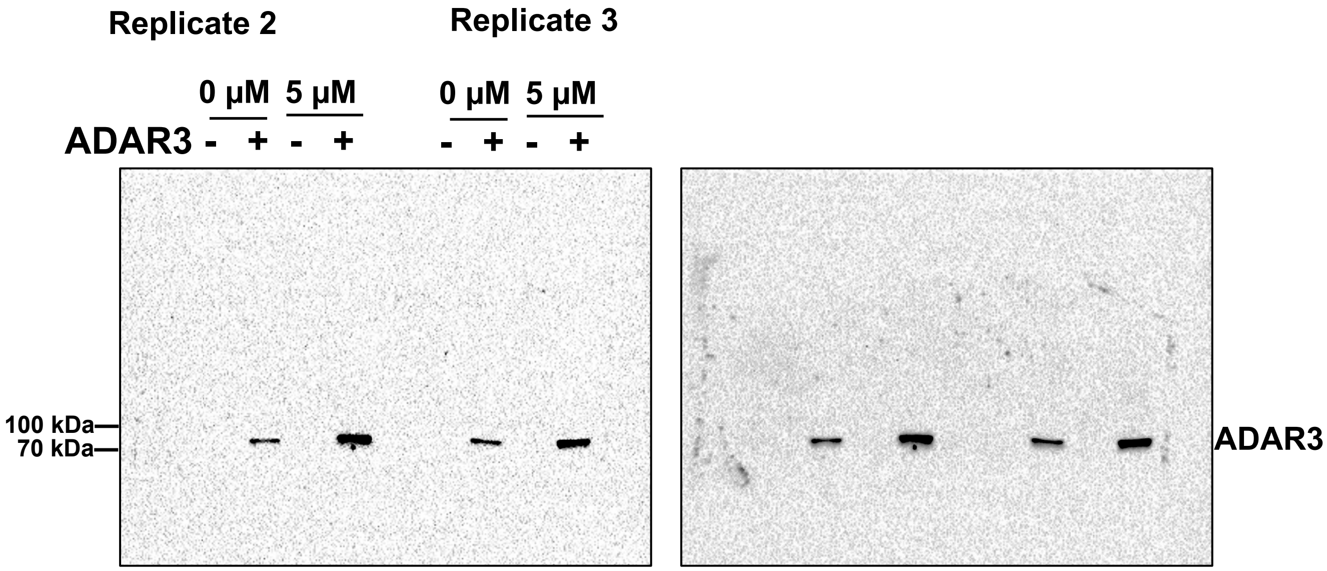

The entire blot images corresponding to Fig 3c The blots were cut at 55 kDa in order to develop using antibody to ADAR3. Two exposures of the ADAR3 blots are shown.

Figure S3a

Replicate 1

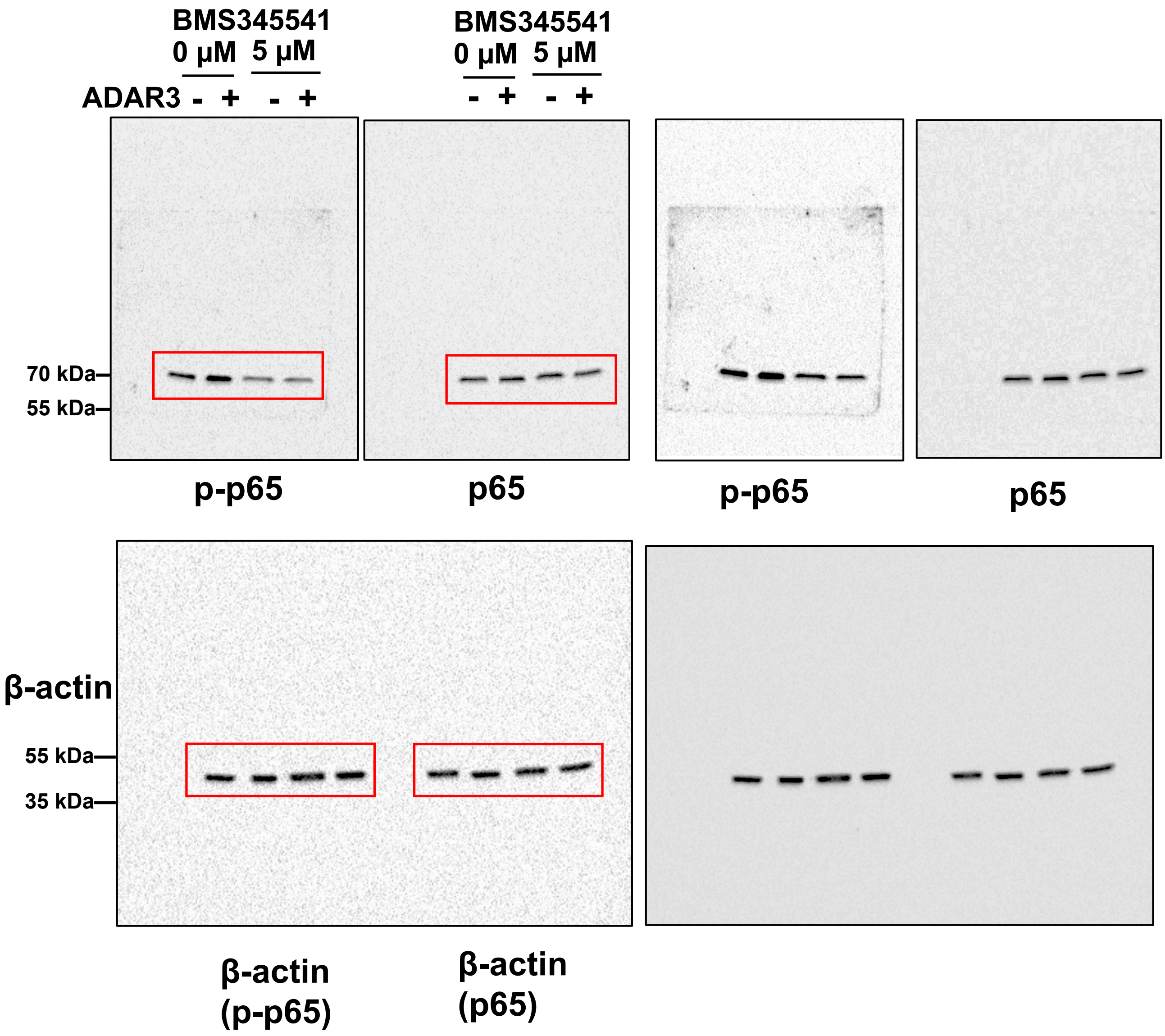

The entire blot images corresponding to Fig S3a. The blots were cut at 55 kDa in order to develop using antibody to p65 or p-p65 and β-actin. Two exposure of each blot is shown.

Figure S3

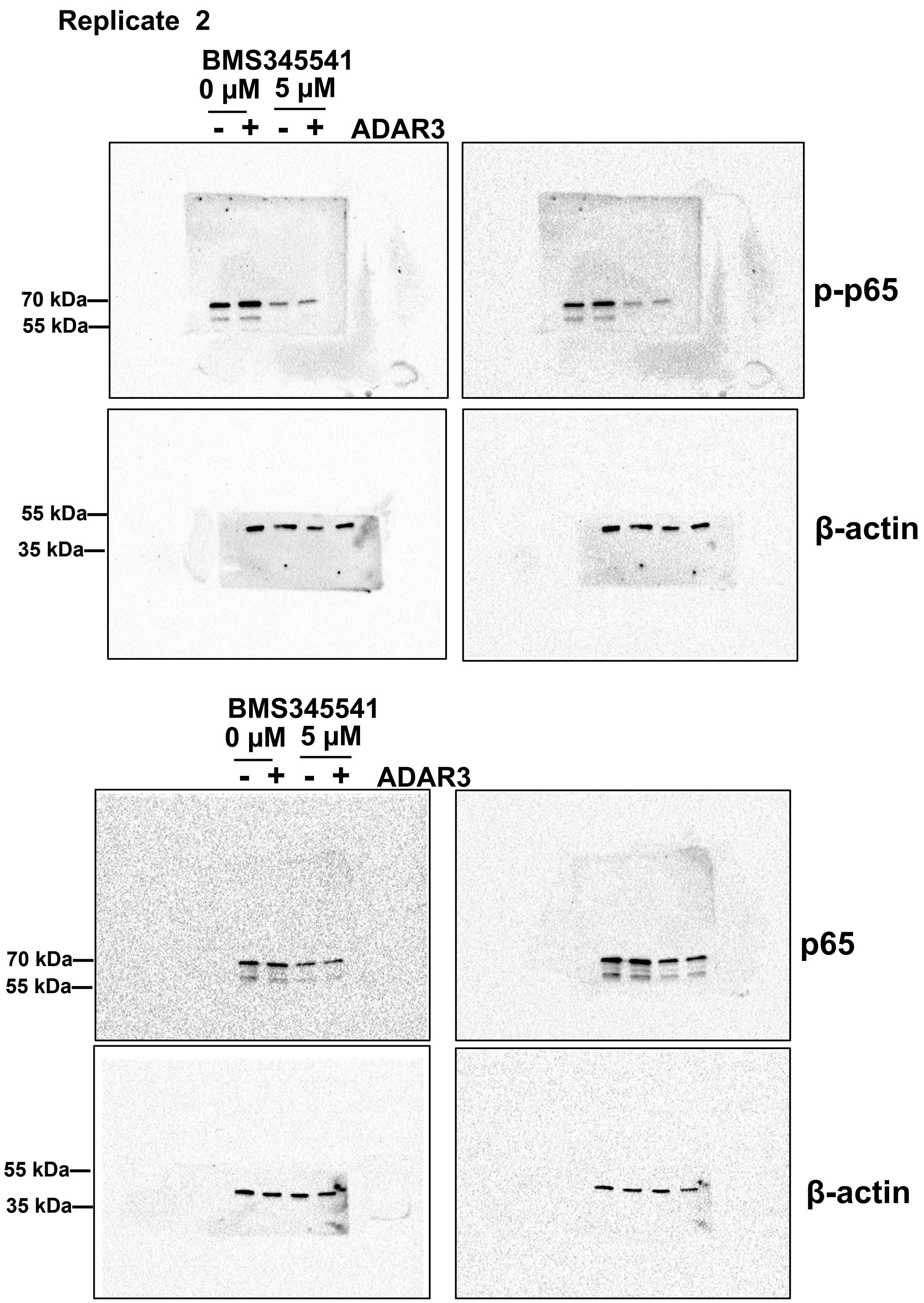

The entire blot images images corresponding to Fig S3a. The blots were cut at 55 kDa in order to develop using antibody to p65 or p-p65 and β-actin. Two exposures of each blot are shown.

Figure S3

Replicate 3

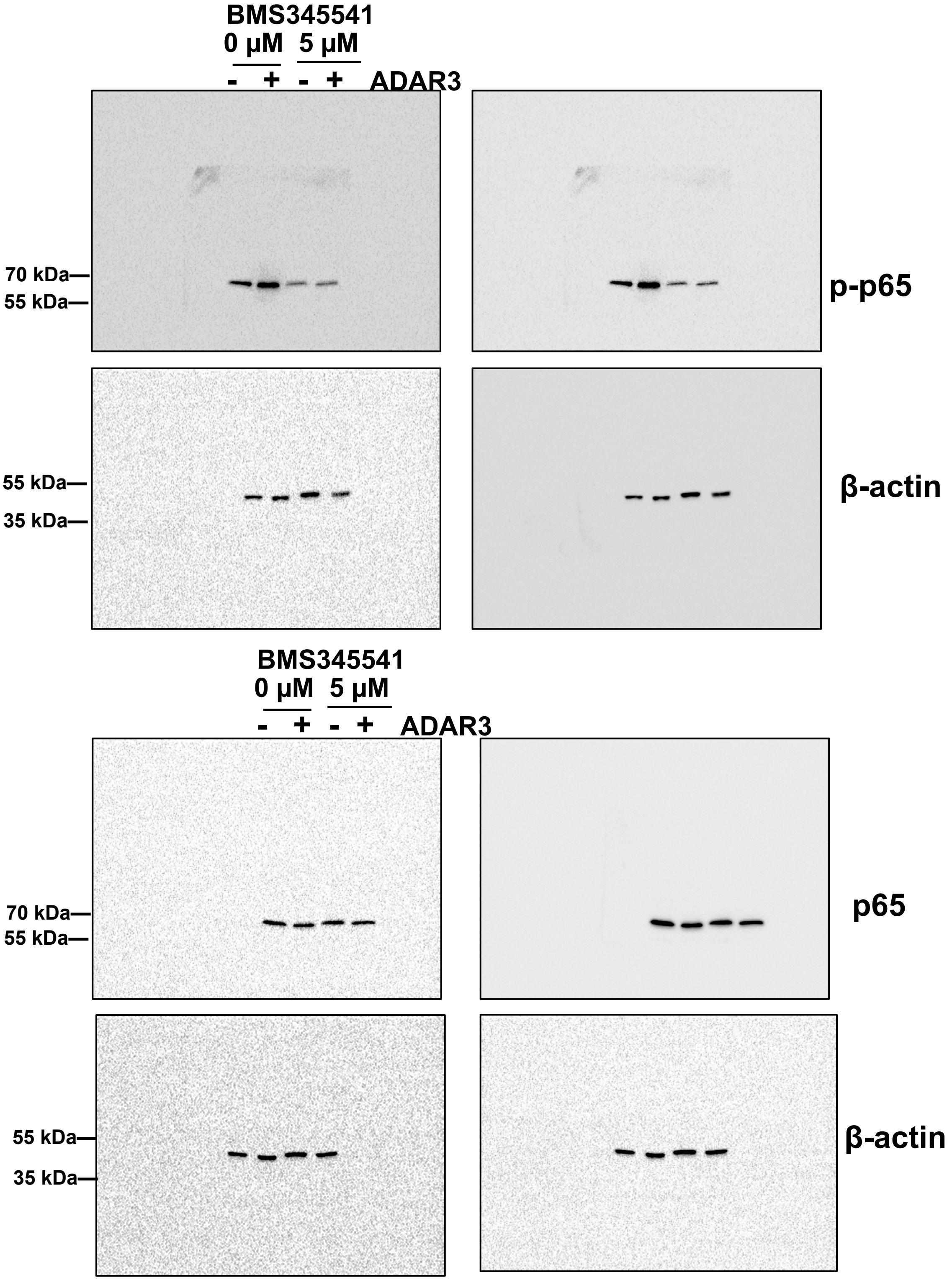

The entire blot images corresponding to Fig S3a. The blots were cut at 55 kDa in order to develop using antibody to p65 or p-p65 and β-actin. Two exposures of each blot are shown.

Figure S3

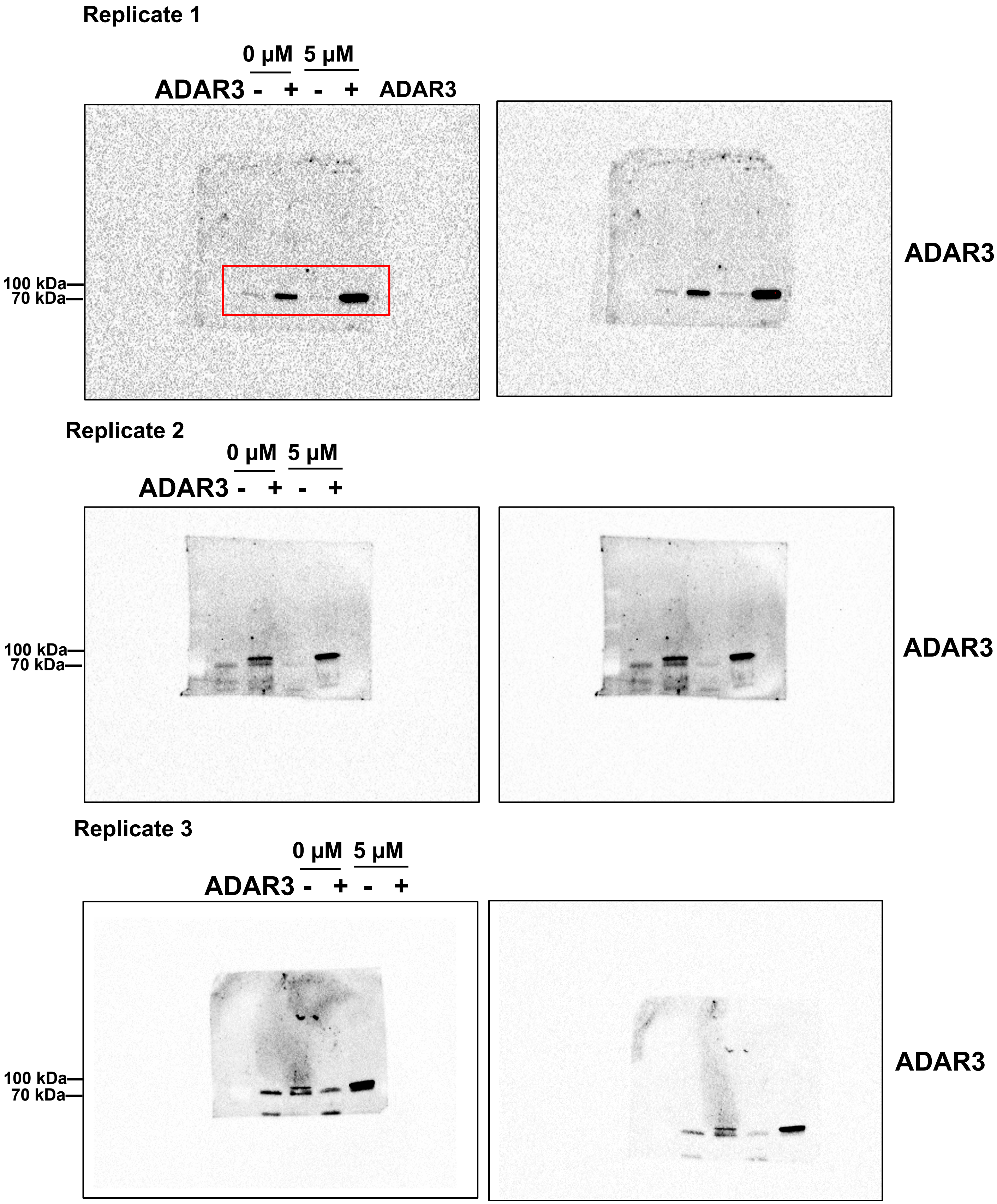

The entire blot images images corresponding to Fig S3a. The blots used for developing p-p65 for each replicate (on previous figure) was reprobed with ADAR3 antibody. Two exposures of each blot are shown.

Figure 4a

Replicate 1

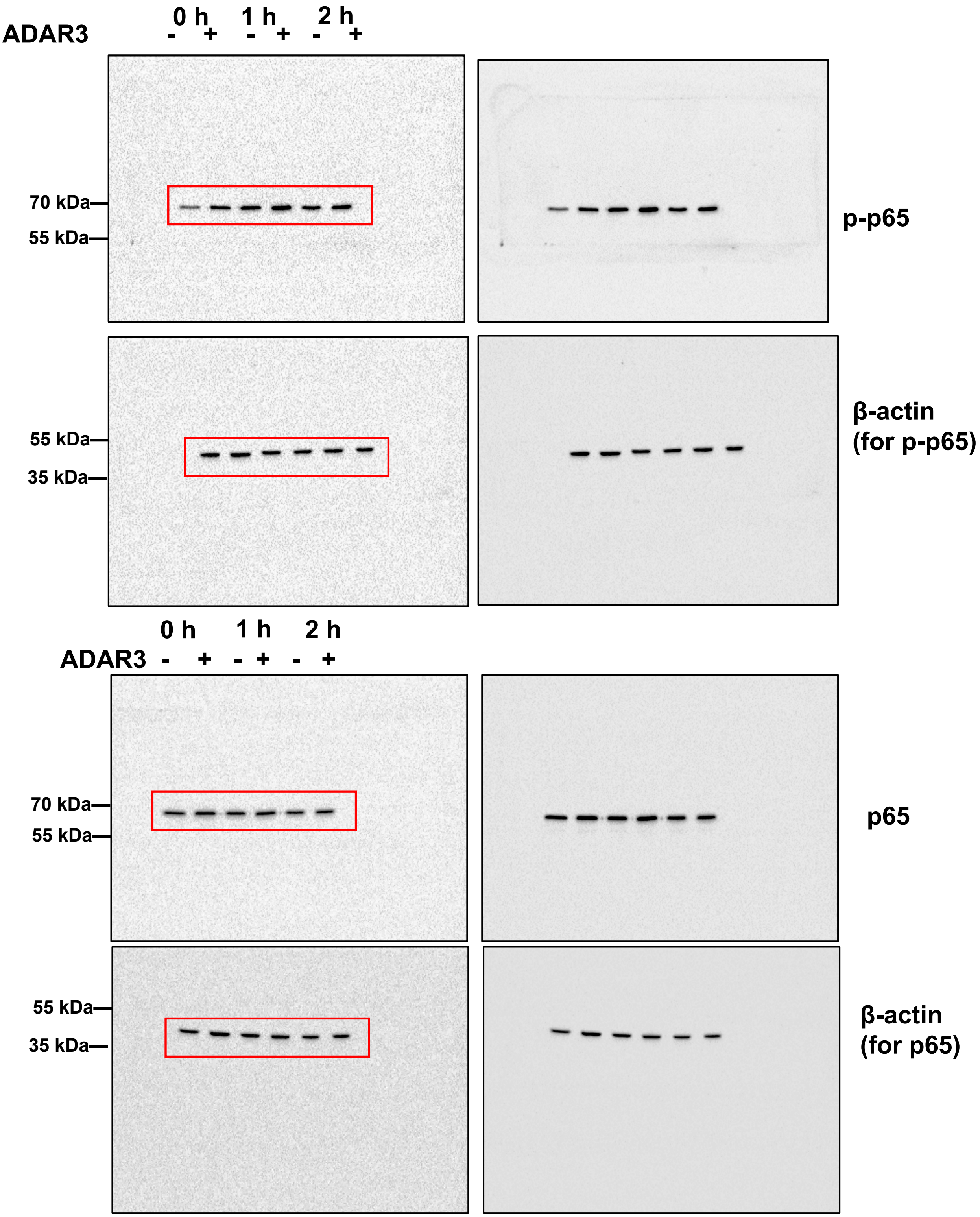

The entire blot images corresponding to Fig 4a are shown. The blots were cut at 55kDa to develop using p65 or p-p65 and β-actin antibodies. Two exposures of each blot are included. Samples are loaded in the same order in all blots.

Figure 4a

Replicate 2

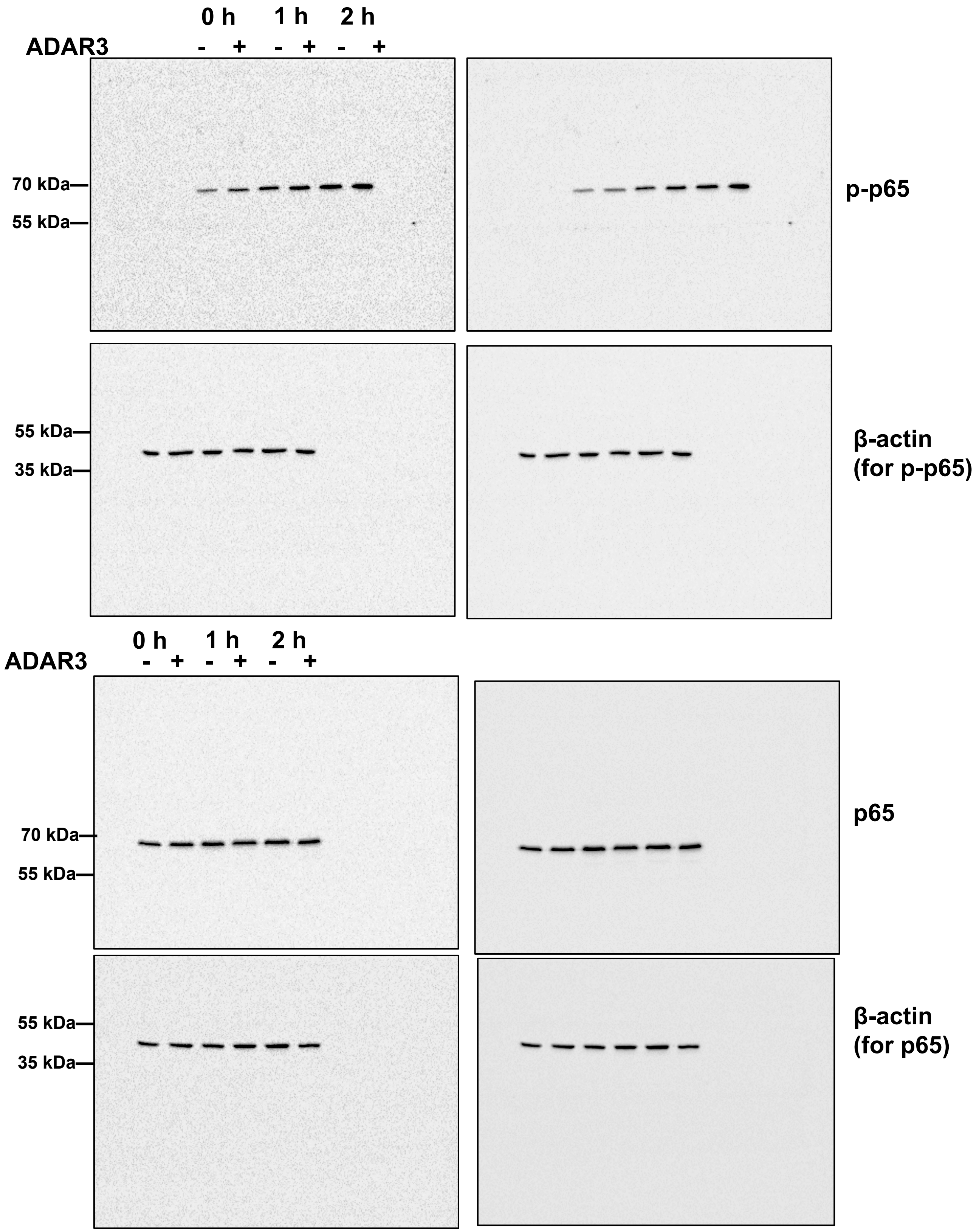

The entire blot images corresponding to Fig 4a are shown. The blots were cut at 55kDa to develop using p65 or p-p65 and β-actin antibodies. Two exposures of each blot are included. Samples are loaded in the same order in all blots.

Figure 4a

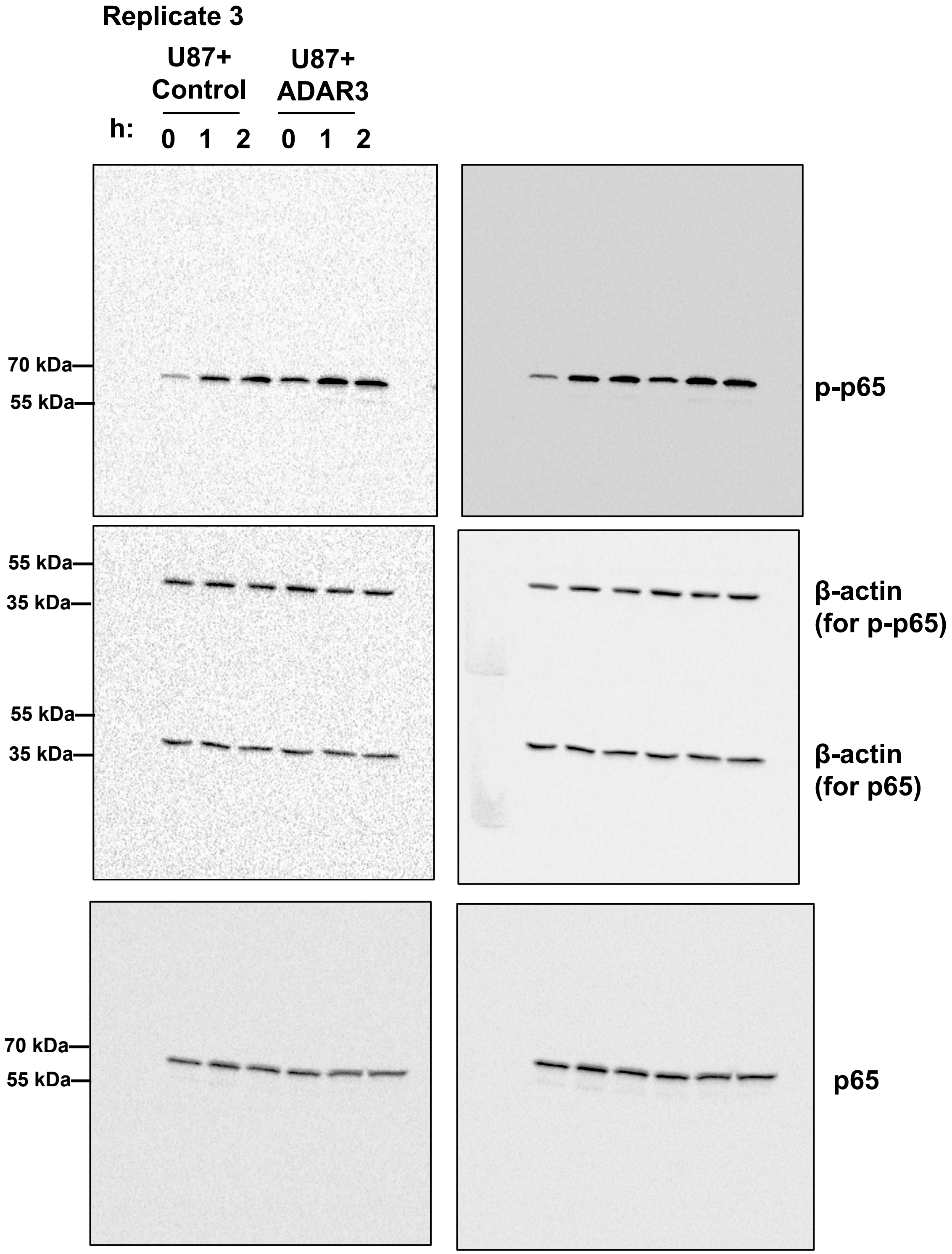

The entire blot images corresponding to Fig 4a are shown. The blots were cut at 55kDa to develop using p65 or p-p65 and β-actin antibodies. Two exposures of each blot are included. The β-actin blots corresponding to p65 (lower) and p-p65 (upper) were imaged together.

Figure 4a

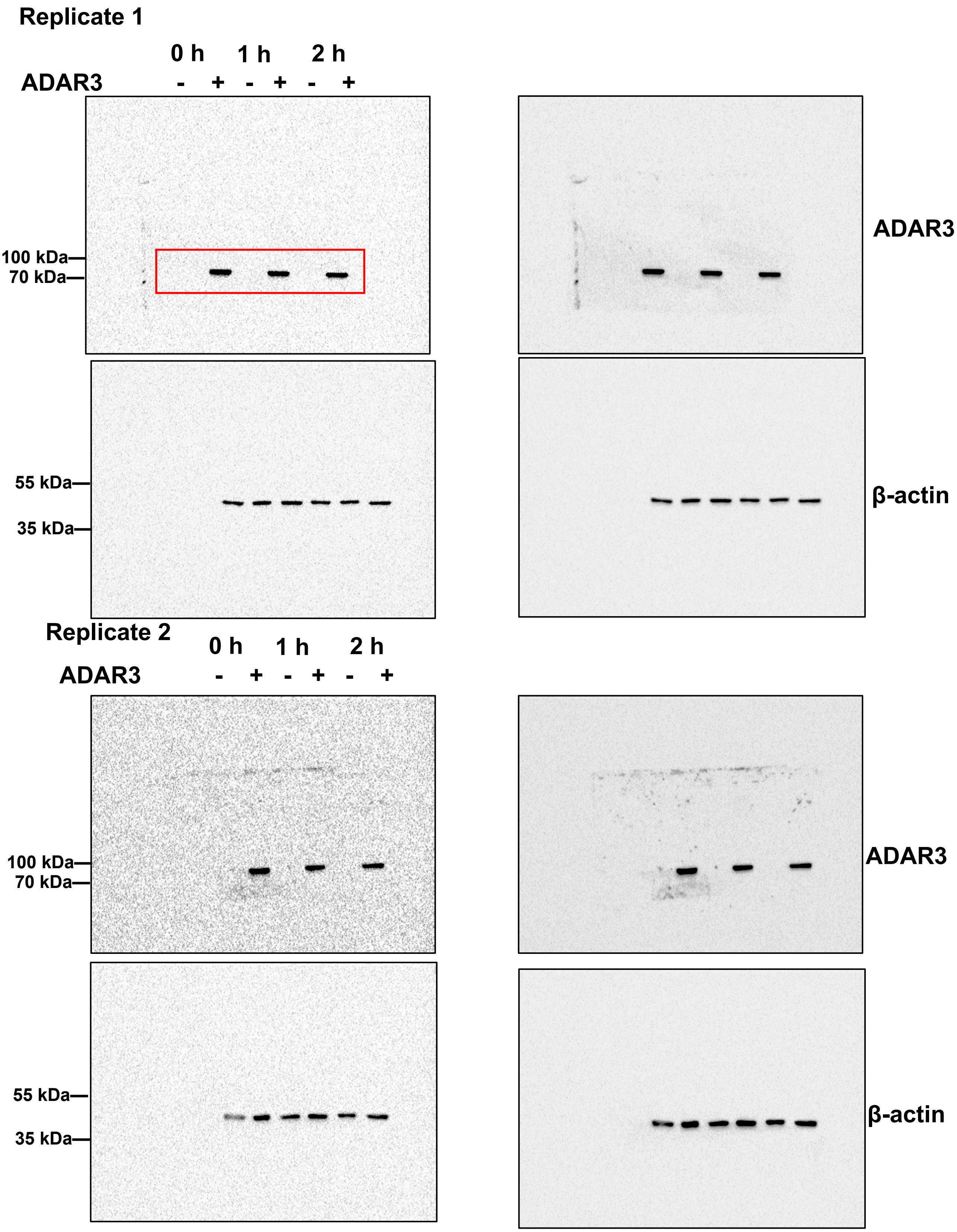

The entire blot images corresponding to Fig 4a are shown. The blots were cut at 55kDa to develop ADAR3 and β-actin antibodies. Two exposures of each blot are included.

Figure 4a

Replicate 3

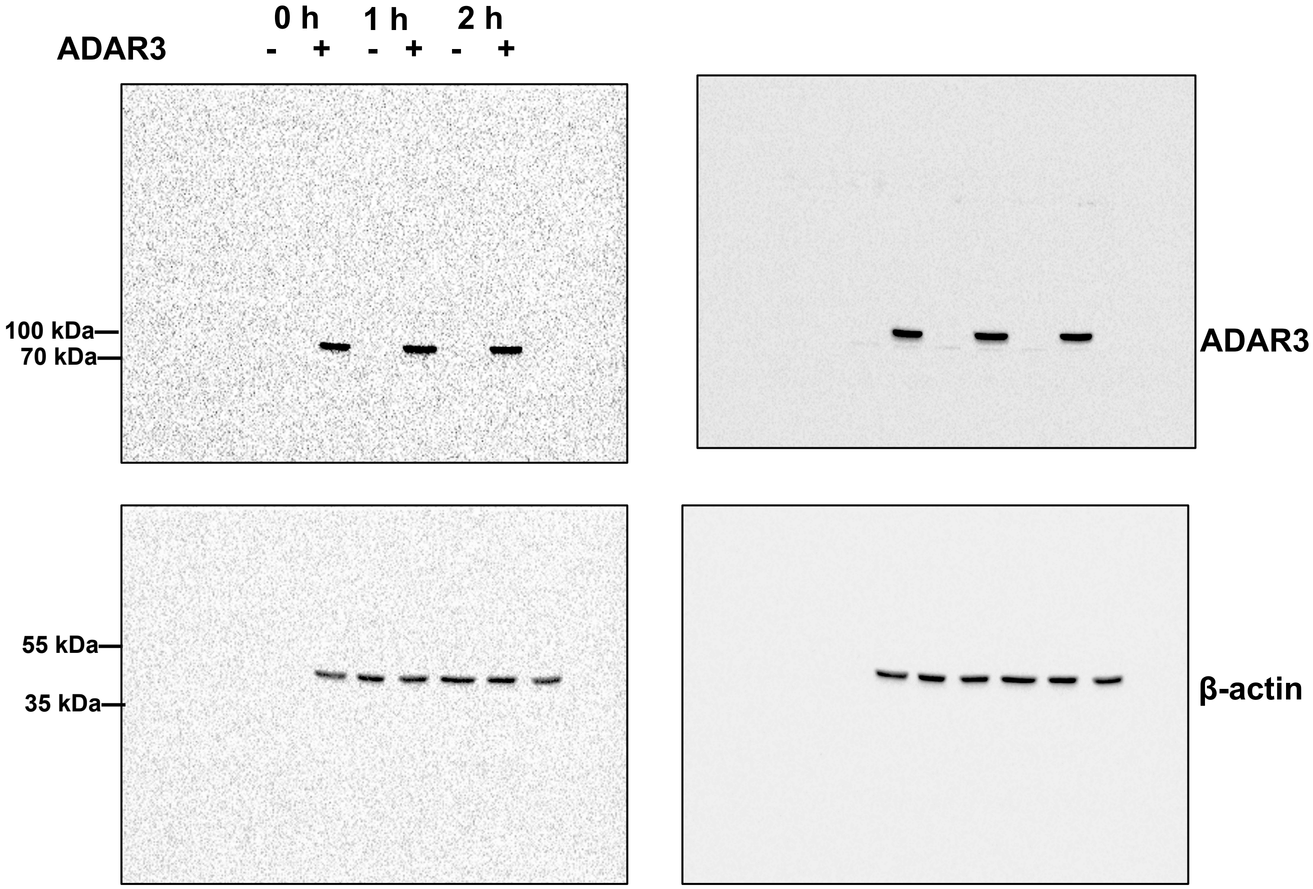

The entire blot images corresponding to Fig 4a are shown. The blots were cut at 55kDa to develop ADAR3 and β-actin antibodies. Two exposures of each blot are included.
